# MyD88/CD40 Genetic Adjuvant Function in Cutaneous Atypical Antigen-Presenting Cells Contributes to DNA Vaccine Immunogenicity

**DOI:** 10.1371/journal.pone.0164547

**Published:** 2016-10-14

**Authors:** Matthew R. Collinson-Pautz, Kevin M. Slawin, Jonathan M. Levitt, David M. Spencer

**Affiliations:** 1 Interdepartmental Program in Translational Biology and Molecular Medicine, Baylor College of Medicine, Houston, TX, United States of America; 2 Department of Pathology and Immunology, Baylor College of Medicine, Houston, TX, United States of America; 3 Bellicum Pharmaceuticals, Houston, TX, United States of America; Mie University Graduate School of Medicine, JAPAN

## Abstract

Therapeutic DNA-based vaccines aim to prime an adaptive host immune response against tumor-associated antigens, eliminating cancer cells primarily through CD8^+^ cytotoxic T cell-mediated destruction. To be optimally effective, immunological adjuvants are required for the activation of tumor-specific CD8^+^ T cells responses by DNA vaccination. Here, we describe enhanced anti-tumor efficacy of an *in vivo* electroporation-delivered DNA vaccine by inclusion of a genetically encoded chimeric MyD88/CD40 (MC) adjuvant, which integrates both innate and adaptive immune signaling pathways. When incorporated into a DNA vaccine, signaling by the MC adjuvant increased antigen-specific CD8^+^ T cells and promoted elimination of pre-established tumors. Interestingly, MC-enhanced vaccine efficacy did not require direct-expression of either antigen or adjuvant by local antigen-presenting cells, but rather our data supports a key role for MC function in “atypical” antigen-presenting cells of skin. In particular, MC adjuvant-modified keratinocytes increased inflammatory cytokine secretion, upregulated surface MHC class I, and were able to increase *in vitro* and *in vivo* priming of antigen-specific CD8^+^ T cells. Furthermore, in the absence of critical CD8α^+^/CD103^+^ cross-priming dendritic cells, MC was still able to promote immune priming *in vivo*, albeit at a reduced level. Altogether, our data support a mechanism by which MC signaling activates an inflammatory phenotype in atypical antigen-presenting cells within the cutaneous vaccination site, leading to an enhanced CD8^+^ T cell response against DNA vaccine-encoded antigens, through both CD8α^+^/CD103^+^ dendritic cell-dependent and independent pathways.

## Introduction

Though once nothing more than background noise, immunotherapies have jumped to the forefront of cutting-edge medicine in recent years, treating a variety of diseases including autoimmunity, atopic disorders, and malignancies. In particular, therapeutic cancer vaccines have garnered much attention, theorizing that the generation of *de novo* immune responses to tumor-specific targets could exploit the full and complex breadth of cell types and secreted factors of the immune system to combat malignant disease [1].

Recent clinical trials of cancer vaccines have supported their potential; however, the results have been modest in most cases and key questions remain to be answered at both the bench and bedside [1,2]. Determining optimal combinations of antigens (Ags), vector design, dose, scheduling, and proper adjuvants remain amongst the largest challenges [1,3]. The ideal therapeutic cancer vaccine should potentiate vigorous professional Ag-presenting cell (APC) activation, along with Ag presentation, to achieve robust T cell priming [3,4]. Due to its versatility and relatively low cost, DNA-based vaccine approaches were introduced in the early 1990s, to modulate humoral and cellular immunity, and extensive research to increase efficacy has followed, in particular through the design of novel immunological adjuvants [4,5]. In addition to co-injection of soluble adjuvants, DNA vaccines can also substitute genetically encoded, immune modulatory components into the vaccine cocktail, such as cytokines (e.g., GM-CSF), chemokines, and immune stimulatory signaling molecules (e.g., CD80), allowing for extended production of adjuvant *in situ*, typically in the skin or muscle [6–9].

Previously, our lab described the development of a synthetic ligand-inducible MyD88/CD40 (“MC”) fusion protein, comprising signaling elements from MyD88 and CD40, two FKBP12-based ligand-binding domains, and a myristoylation membrane localization domain, as an adjuvant for cancer vaccines [10]. In the presence of the small molecule chemical inducer of dimerization, rimiducid (rim), MC oligomerization within dendritic cells (DCs) led to simultaneous activation of innate toll-like receptor (TLR) and adaptive CD40 signaling pathways, resulting in upregulation of activation markers (e.g., CD83, CD80/86, MHC) and secretion of Tc1-polarizing cytokines, such as IL-12p70. Initial use of MC as a genetic adjuvant in an autologous, *ex vivo* DC vaccine demonstrated that MC enhanced cytotoxic T cell (CTL) responses against tumors *in vivo*. Further research showed that MC could be used in “off-the-shelf” vaccines using viral-mediated delivery [11], thus, supplanting the need for *ex vivo DC* manipulation; however, efficacy was hindered by pre-existing host anti-viral-vector immunity. These results illustrated not only the need for further development of “off-the-shelf” methodologies, but also for a better understanding of how genetic adjuvants like MC function when expressed in a broad set of cell types at the vaccination site. Importantly, while the skin is recognized as a barrier tissue that influences innate and acquired immune responses, little work has been done to investigate how expression of adjuvants, such as MC in skin cell subsets, not typically considered for their immune-modulatory functions, contribute to adjuvant-enhanced DNA vaccine-mediated immune response.

Herein, we report a novel application for MC adjuvant to enhance the efficacy of DNA vaccines delivered by *in vivo* electroporation (EP). MC-enhanced EP vaccination improved priming and propagation of anti-tumor Ag (anti-TAg) T cell responses in therapeutic mouse models of melanoma and lymphoma. *In vitro*, expression of MC in non-hematopoietic cutaneous cell types (e.g., keratinocytes) imparted an atypical APC phenotype, enabling Ag-presentation to naïve T cells, upregulation of costimulatory molecules, and secretion of Tc1-cytokines. Furthermore, we describe the *in vivo* action of MC adjuvant when expressed in cutaneous non-hematopoietic cell types present at the site of vaccine administration, revealing both a CD8α^+^/CD103^+^ DC-dependent and independent mechanism, overall demonstrating an important immunological contribution of MC signaling in atypical APCs to the vaccine-mediated augmentation of anti-TAg cytotoxic T cell responses.

## Materials and Methods

### Mice, cell lines, recombinant plasmids, and dimerizer drug

6 to 8-week-old female C57BL/6 and BALB/c mice were purchased from the Center for Comparative Medicine at Baylor College of Medicine (BCM; Houston, TX) or the Jackson Laboratory (Bar Harbor, ME). 6-week old female C57BL/6-Tg(TcraTcrb)1100Mjb/J (a.k.a., OT-1) and 7-week-old B6.129S(C)-*Batf3*^*tm1kmm*^/J (a.k.a. *Batf3*^-/-^) mice were purchased from the Jackson Laboratory. All mice were housed in pathogen-free conditions in either the Transgenic Mouse Facility (TMF) at BCM, the Center for Laboratory Animal Medicine and Care at University of Texas Health Science Center, or Bellicum Pharmaceuticals’ Animal Facility. All animal studies were performed in accordance with the guidelines set by the National Institutes of Health (NIH Publication No. 85–23, revised 1996) and under protocols approved by the respective Institutional Animal Care and Use Committees (IACUCs) at each aforementioned facility (Protocol nos.: BCM, AN-1428; UT-HSC, HSC-AWC-13-120; Bellicum, 2016–002). For long-term tumor and survival studies, a tumor volume of 1.5 cm^3^ was used as a surrogate endpoint, and animals were monitored every 2–3 days throughout the course of the experiment for suffering and distress. For all other animal experiments, including those examining immune responses to vaccination in naïve or tumor bearing mice, animals were terminated at a predetermined endpoint, based on the expected peak immune response, usually 1–10 days following the last vaccination. At the specified endpoints, animals were euthanized by isoflurane overdose followed by cervical dislocation. Animals were anesthetized by isoflurane during all subQ injections (e.g., EP vaccinations) to minimize stress and discomfort.

HEK293, EG.7, and NIH3T3 cell lines were purchased from ATCC (Manassas, VA) and cultured following vendor’s guidelines for each respective cell line. MPEK-BL6, a spontaneously transformed keratinocyte cell line derived from C57BL/6 normal tissue, was purchased from CELLnTEC (Bern, Switzerland), and cultured in CntPrime Media following vendor’s guidelines.

For all vaccine vectors, a modified pCDNA3.1 backbone (Thermo Fisher Scientific, Waltham, MA), termed pCDNA.mini, was used. Specifically, the f1 phage origin of replication, SV40 promoter, and NeoR/KanR antibiotic-resistance cassettes were removed, leaving the remaining features of pCDNA3.1 intact. Standard PCR and restriction cloning techniques were used to generate plasmids. The chicken ovalbumin (Ova) gene, used in “OVA” and “MC.OVA” plasmids, was derived from the protein sequence of NCBI Accession no. CD02059. The LacZ gene used in LacZ is as previously described [10]. The MyD88/CD40 (MC) genetic adjuvant sequence is as previously reported [11]. Bicistronic vectors, MC.OVA and MC.OVA.miR142T, contain a P2A polypeptide separation sequence derived from the *Porcine teschovirus* 2A sequence [12]; 5’- ggctCCGGAGCTACTAACTTCAGCCTGCTGAAGCAGGCTGGAGACGTGGAGGAGAACCCTGGACCT-3’. The P2A sequence was positioned between the MC and OVA genes. Four repeats of the miR142-3p target sequence (miR142T) [13], were cloned into the 3’-UTR of the parental pCDNA.mini backbone to generate pCDNA.mini.miR142T backbone. The miR142T sequences were placed between the stop codon and poly A, as follows (**Bold** indicates miR142-3p target sequence): 5’- TTAAGAGCGGCCGCTCTAGAG**TCCATAAAGTAGGAAACACTACA**CGATGATC**TCCATAAAGTAGGAAACACTACA**ACGGTCACGTAT**TCCATAAAGTAGGAAACACTACA**TCACCTAG**TCCATAAAGTAGGAAACACTACA**TCAGTCGAGCACTCATACTCTAGAGTTTAAACCGCTGATCAGCCTCGA-3’. For validation of miR142T restricted expression, the gene encoding the transmembrane protein human prostate-specific-membrane antigen (PSMA, NCBI Accession no. AK312366.1) was cloned into pCNDA.mini.miR142T to generate PSMA.miR142T and MC.PSMA.miR142T. Following *in vitro* validation of pCDNA.mini.miR142T, OVA.miR142T and MC.OVA.miR142T were generated by restriction cloning of the gene encoding OVA into PSMA.miR142T and MC.PSMA.miR142T to generate OVA.miR142T and MC.OVA.miR142T.

The dimerizer drug, rimiducid [14], was provided by Bellicum Pharmaceuticals (Houston, TX). For *in vitro* assays, rimiducid was dissolved in ethanol at a 100 μM stock concentration. For *in vivo* administration, 6.25 μL of 5 mg/mL rimiducid in 25% Kolliphor® HS15 (Solutol HS15), was diluted in sterile saline to a total volume 100 μL per mouse and a final dose of 1.25 mg/kg was injected intraperitoneal (IP).

### *In vivo* electroporation and vaccination

*In vivo* electroporation (EP) was performed with an Ichor Medical Systems TriGrid Instrument (San Diego, CA). All plasmid DNA used for *in vivo* applications was prepared using an endotoxin-free (EndoFree) Giga-prep kit (Qiagen, Hilden, Germany), and stock plasmid solutions were diluted in sterile PBS for a total volume of 50 μL per injection just prior to subcutaneous (subQ) administration. Animals were anesthetized using isoflurane during all procedures. Animals were shaved on their hind limbs and dorsal surfaces. 50 μL plasmid DNA (pDNA), 25–50 μg per injection, was injected intradermal (ID) into the flank (TriGrid EP device) using a 28.5-gauge needle. Immediately following injection, needle array probes were placed over the “bubble” formed by the injection bolus and electrical stimulation was applied. Electrical pulses were pre-set for the TriGrid system. Initial vaccinations and subsequent booster vaccinations were administered on alternating, contralateral flanks.

For vaccinations using OVA as the Ag in naïve mice, animals received two 25 μg doses of vaccine 14–21 days apart, each administered on contralateral flanks. Animals in groups receiving dimerizer were injected IP with 1.25 mg/kg rim 24 hours after each vaccination.

### Production of lentivirus, transduction and selection of stable cell lines

The pCDH cDNA expression and lentivirus backbone was obtained from System Biosciences (Mountain View, CA). The pCDH backbone contains both GFP and puromycin resistance genes. The following primers were used to amplify the Ova gene with a 5’ EcoRI and 3’ NotI restriction site to clone into the pCDH backbone: 5’ forward primer–(5’–GAATTCgccaccatgggctccatc– 3’); 3’ reverse primer–(5’—gcggccgcttaaggggaaacacatctgccaaagaagag– 3’). Similarly, the MC.OVA gene was amplified using the following primers to generate a PCR fragment with 5’ EcoRI and 3’ NotI restriction sites for cloning into the pCDH lentiviral backbone: 5’ forward primer–(5’–gtacgtgaattcgccaccatggggagtagcaag– 3’). Lentivirus was produced by transfection of 10 μg pCDH vector (empty, OVA, or MC.OVA), 10 μg VSV-G plasmid, and 10 μg Gag-Pol plasmid in 293T cells using GeneJuice Transfection reagent (Millipore, Billerica, MA). Viral supernatants were collected after 48 hours and filtered through a 0.45 μm syringe filter. Parental NIH3T3 and MPEK cell lines were transduced overnight with 2 mL of viral supernatants and 4 ng/mL polybrene (Sigma Aldrich). Starting one week after transduction, cells positive for transgene integration were selected with 1 μg/mL puromycin for two weeks. The percent of transgene^+^ cells was determined by GFP expression as measured by flow cytometry.

### Vaccinations with MPEK cell lines

C57BL/6 mice received injections of 5 x 10^5^ MPEK stable cells (Negative control, OVA, or MC.OVA) two times 21 days apart. Animals in groups receiving dimerizer were injected IP with 1.25 mg/kg rim 24 hours after each injection. Spleens were isolated 7 days after the second injection and analyzed for SIINFEKL-specific IFN-γ-secreting splenocytes by ELISpot (see method description below).

### IFN-γ ELISpot assays

Spleens were dissected from mice 6–8 days following final vaccination. In one experiment, mice were vaccinated with a single 50-μg dose of pDNA encoding GFP, LacZ, or LacZ + EP. In other experiments, naïve or tumor-bearing mice were vaccinated at least twice with 25 μg pDNA dose on contralateral flanks followed by EP. Single-cell suspensions of splenocytes were made by mechanical dissociation and red blood cells were removed by lysis with ACK buffer (Lonza, Basel, Switzerland). 3 x 10^5^ splenocytes per well were seeded into 96-well mixed-cellulose esters membrane plates (Millipore) that had been coated overnight with 5 μg/mL anti-mouse-IFN-γ capture antibody (Cat# 551216, BD Biosciences, San Jose, CA) in calcium bicarbonate buffer (Sigma-Aldrich, St Louis, MO) and then blocked with a sterile 2% BSA solution in PBS. Splenocytes were stimulated overnight with 2.5 μg/mL of either OVA_257-264_ (SIINFEKL) H2-K^b^-restricted peptide, β-gal_497-504_ (ICPMYARV) H2-K^b^-restrictued peptide, irrelevant TRP2_180-188_ (SVYDFFVWL) peptide (Genemed Synthesis, San Antonio, TX), media alone, or 5 ng/mL PMA + 500 ng/ml Ionomycin (positive control) overnight at 37°C in 5% CO_2_. Cells were removed from plates, and plates washed with PBS + 0.5% Tween-20. Plates were incubated with 100 μL per well of 1 μg/mL biotin-conjugated anti-mouse-IFN-γ detection antibody (Cat# 554410, BD Biosciences) in 2% BSA solution for 1 hour at 37°C. Plates were again washed with PBS + 0.5% Tween-20. 100 μL of ExtrAvidin–alkaline phosphatase (Sigma-Aldrich, St Louis, MO) diluted 1:5000 in PBS + 2% BSA was added to each well and incubated for 30 mins at RT. Plates were again washed with PBS + 0.5% Tween-20. SIGMAFAST BCIP/NBT (Sigma-Aldrich) substrate was dissolved per the manufacturer’s guidelines and 100 μL added per well. Wells were allowed to develop for 5–15 minutes. Membranes were sent to ZellNet Consulting (Fort Lee, NJ) for spot counting and analysis.

### Dextramer and tetramer analysis

Spleens of vaccinated or control mice were isolated and single-cell suspensions made as outlined above. For dextramer analysis, 1 x 10^6^ splenocytes in FACs buffer (PBS + 5% FBS) were then stained with 5 μL SIINFEKL-H2-K^b^-PE dextramer (Immudex, Copenhagen, Denmark) for 10 minutes at RT in the dark. After 10 minutes, a staining cocktail of anti-mouse-CD3-FITC (BioLegend) and anti-mouse-CD8α-PerCP/Cy5.5 (Cat# 100734, BioLegend) was added to each sample. Samples were incubated for another 20 minutes at 4°C. For tetramer staining, 1 x 10^6^ splenocytes in FACs buffer were stained with 1 μL SIINFEKL-H2-K^b^-PE tetramer (MHC Tetramer Production Core, BCM, Houston, TX), anti-mouse-CD3-FITC, anti-mouse-CD4-APC-Cy7 (Cat# 100414, BioLegend), and anti-mouse-CD8α-PerCP/Cy5.5 for 1 hour at 4°C. Stained cells were analyzed on a Gallios flow cytometer (Beckman Coulter).

### Western blot

HEK-293 cells were transfected with GeneJuice (Millipore) with 1 μg of GFP, OVA, or MC.OVA plasmid. After 24 hours, cells were harvested, lysed in Peirce RIPA buffer with 1x Halt Protease Inhibitor Cocktail (ThermoFisher Scientific) for 30 mins on ice. Lysates were diluted in Laemmli loading buffer (BioRad) with β-mercaptoethanol (Sigma) then denatured at 95°C for 10 minutes. Samples were run on a 10% polyacrylamide gel and transferred to a PVDF membrane using the iBlot 2 dry transfer system (ThermoFisher Scientific). Membranes were probed with primary monoclonal mouse anti-chicken ovalbumin antibody (Sigma, clone OVA-14, 1:250 dilution) and secondary goat-anti-mouse IgG HRP conjugated antibody (ThermoFisher Scientific, cat no. 31461, 1:500 dilution). Probing for β-actin as a loading control was done using primary rabbit polyclonal anti-β-actin (ThermoFisher Scientific, cat no. PA1-16889, 1:1000 dilution) and secondary goat-anti-rabbit IgG HRP (ThermoFisher Scientific, cat no. 31436, 1:2000 dilution). Blocking, staining, and washing were done overnight using the iBind Flex Western device and reagents following the manufacturers protocol (ThermoFisher Scientific). Bands were visualized using Super Signal West Femto (ThermoFisher Scientific) and a Gel Logic 6000 Pro (Carestream). Band densities were measured using ImageJ freeware (NIH, Bethesda, MD).

### NF-κB secreted alkaline phosphatase (SEAP) assays

HEK-293 or IC21 cells were transfected using GeneJuice (Millipore) with 1 μg of NF-κB-SEAP reporter plasmid and 1 μg of GFP, PSMA, PSMA.miR142T, MC.PSMA, MC.PSMA.miR142T, OVA, OVA.miR142T, MC.OVA, or MC.OVA.miR142T. 24 hours later, cells were harvested, washed, and seeded in a 96-well flat-bottom plate with increasing concentrations of rim. After another 24 hours, supernatants were heat-inactivated at 68°C for 1 hour and analyzed for SEAP activity. Briefly, to measure SEAP activity, heat-inactivated supernatants were mixed 1:1 with 4-methylumbelliferal-phosphate (MUP) substrate solution (1 mM MUP in 2M diethanolamine) in a black 96-well plate and incubated at 37°C for 1–18 hours depending on signal strength. Fluorescence was measured with 355 nm excitation and 460 nm emission filters using a POLARstar plate reader (BMG Labtech, Ortenberg, Germany).

### ELISA and multiplex cytokine/chemokine assays

Splenocytes from vaccinated or naïve mice were isolated as described above. Single cell suspensions were seeded at 3 x 10^5^ cells/well in a 96-well flat-bottom plate. Splenocytes were stimulated overnight with 2.5 μg/mL of either OVA_257-264_ (SIINFEKL) H2-K^b^-restricted peptide, β-gal_497-504_ (ICPMYARV) H2-K^b^-restricted peptide, irrelevant TRP2_180-188_ (SVYDFFVWL) H2-K^b^-restricted peptide, media alone, or 5 ng/mL phorbol 12-myristate 13-acetate (PMA) + 500 ng/mL Ionomycin (positive control) overnight at 37°C in 5% CO_2_. Supernatants were collected after 24 hours and cytokine concentrations were quantitated either using a standard mouse ELISA assay (IFN-γ) (Millipore) or murine Cytokine/Chemokine multiplex kit (Millipore) measuring 25 different analytes, as per the manufacturer’s protocol.

MPEK or NIH3T3 cells were seeded at 2 x 10^5^ cells per well in a 48-well flat-bottom plate in 500 μL culture media. 10 nM rim was added to the indicated samples. Cells were cultured at 37°C in 5% CO_2_. Supernatants were collected after 48 hours and analyzed for cytokine/chemokine levels using a murine multiplex kit (Millipore) measuring 25 different analytes, as per the manufacturer’s instructions.

### *In vivo* CTL assay

6 to 8-week-old female C57BL/6 mice were vaccinated by EP with two 25-μg doses of pDNA 20 days apart. 1.25 mg/kg rim was administered IP 24 hours after each vaccination in some groups. 7 days following the final vaccination 1 x 10^7^ dye-labeled and peptide-pulsed syngeneic splenocytes were adoptively transferred. To prepare adoptively transferred splenocytes, initially, spleens from naïve C57BL/6 mice were harvested and single-cell suspensions were made. Cells were then counted and seeded at 1 x 10^7^ cells/well in a 96-well V-bottom plate. Cells were then labeled with CellTrace Violet (CTV) cell proliferation dye (ThermoFisher Scientific, Waltham, MA) at either a high (Hi) concentration (5 μM) or low (Lo) concentration (0.5 μM) for 25 minutes at 37°C, per the manufacturer’s recommendations. Cells were then washed and “Hi” CTV-labeled cells were pulsed with 5 μg/mL OVA_257-264_ (SIINFEKL) H2-K^b^-restricted peptide, and the “Lo” CTV-labeled cells were pulsed with 5 μg/mL β-gal_497-504_ (ICPMYARV) H2-K^b^-restricted peptide for 1 hour at 37°C in RPMI + 10% FBS + 1% Pen/Strep. Cells were then washed with complete RPMI media and counted. Hi and Lo cell populations were mixed at a 1:1 ratio. 1 x 10^7^ cells total per mouse were then injected IV. Adoptively transferred cells were allowed to circulate for 7 hours. After 7 hours, spleens were removed and specific lysis of target, SIINFEKL-pulsed, cells was calculated based on previously reported methods [15]. Briefly, ratios of “Hi” to “Lo” labeled cells were determined by flow cytometry. To determine the specific lysis for each sample, the following calculation was used: Percent Specific Lysis = (1 - (Non-Transferred Cells Ratio / Experimental Ratio)) * 100.

### *In vitro* OT-1 T cell proliferation

To isolate OT-1 transgenic CD3^+^CD8^+^ T cells, inguinal lymph nodes and spleens were harvested from 8-week-old female OT-1 transgenic mice (Jackson Laboratory). Single-cell suspensions were made from each tissue and pooled to increase total cell yields from each animal. Negative selection by a magnetic-activated cell sorting (MACs) kit (Miltenyi Biotec, Bergisch Gladbach, Germany) was used to purify CD3^+^CD8α^+^ T cells following the manufacturer’s protocol. Briefly, cells were incubated with biotin-antibody cocktail at 1 x 10^7^ cells / 50 μL total volume (40 μL buffer + 10 μL antibodies) for 5 minutes at 4°C. Labeled cells were then applied to an LS column (Miltenyi Biotec) in a QuadroMACs (Miltenyi Biotec) magnetic separator. Unbound cell fraction, containing CD3^+^CD8α^+^ cells was collected and counted. OT-1 T cells were then labeled with 5 μM CTV dye. 5 x 10^5^ CTV-labeled T cells were seeded in a 96-well round-bottom plate alone or with 5 x 10^5^ MPEK cell lines OVA, MC.OVA, or vector control. Co-cultures were incubated at 37°C in 5% CO_2_ for 60 hours. After 60 hours, cells were harvested from wells and stained with anti-mouse-CD3-AlexaFluor647 (Cat# 100209, eBioscience, San Diego, CA), anti-mouse-CD8α-PE/Cy7 (Cat# 100722, BioLegend) and analyzed by flow cytometry. Live cells within the CD8^+^CD3^+^ gate were measured for proliferation by dilution of CTV dye.

### Therapeutic tumor experiments

C57BL/6 mice were injected subQ with 5 x 10^5^−1 x 10^6^ E.G7-Ova tumor cells. Tumors were allowed to grow for 2–5 days before mice were randomized into treatment groups. In long-term therapeutic studies, mice received two vaccinations with the first given between days 2 and 3 post-tumor injection, followed by a second injection 5–6 days later. Mice were vaccinated by EP (Ichor) with 25 μg of GFP, OVA, MC.OVA, OVA.miR142T, or MC.OVA.miR142T-encoding plasmids. For short-term experiments examining T cells responses in tumor-bearing mice, mice were vaccinated similarly with GFP, OVA, or MC.OVA on days 5 and 11 post-tumor injection, and euthanized on day 19 post-tumor injection, and spleens and tumors resected for further analysis. Administration of 1.25 mg/kg rim IP occurred 24 hours after each vaccination in indicated groups. In long-term B16-OVA tumor experiments, C57BL/6 mice were injected subQ with 2 x 10^5^ B16-OVA tumor cells. Mice were randomized on day 6 and vaccinated on days 6, 12, and 19 post-tumor injection by EP with 25 μg of either GFP, OVA, or MC.OVA ± rim. Tumors were measured by length and width using Vernier calipers. Tumor volume was calculated as; Tumor Volume = 0.5236 × Length × Width^2^ [16].

### Surface marker expression analysis on MPEK and NIH3T3 stable cell lines

NIH3T3 or MPEK stable cell lines were seeded into a 6-well plate. Some wells received 10 nM rim. Cells were incubated at 37°C in 5% CO_2_ for 48–72 hours. Cells were harvested using trypsin, then stained and analyzed for surface expression and upregulation by flow cytometry. NIH3T3 cell lines were stained with anti-mouse-H2-K^q^-AlexaFluor 647 (Cat# 115106), anti-mouse-CD80-PerCpCy5.5 (Cat# 104722), anti-mouse-CD86-APC/Cy7 (Cat# 105030) (BioLegend), and anti-mouse-CD40-PE (Cat# 12-0401-82, eBioscience). MPEK cell lines were stained with anti-mouse-anti-H2-K^b^-PE (Cat# 116508), anti-mouse-CD80-PerCpCy5.5, anti-mouse-CD86-APC/Cy7, and anti-mouse-CD40-PE.

### Statistics and graphs

All statistical analyses were performed using GraphPad Prism software, version 6.07 (GraphPad, La Jolla, CA). Parametric assumptions were made for the data analyzed. An unpaired student’s t-test to calculate two-tailed p-values for comparing two samples was used. For comparison of three or more groups, one-way ANOVA with Tukey or Dunnett’s correction for multiple comparisons was performed. Tumor growth curves were analyzed and compared by two-way ANOVA with repeated measures, correcting for multiple comparisons using the Holm-Šídák method. Statistical outliers were assessed by Dixon’s Q-test and removed. Unless otherwise indicated, error values presented in figures are standard deviation (SD). Survival statistics were analyzed using the log-rank (Mantel-Cox) test. Goodness of fit and correlation curves were analyzed by non-linear regression using a one-phase decay fit to obtain R^2^ values. Heat maps were generated using the online freeware Plot.ly (Montreal, Canada), all other graphs were generated using GraphPad Prism.

Supplemental methods and materials provided in supporting information (S1 Supplemental Methods).

## Results

### Adjuvant MyD88/CD40 enhances DNA vaccine-mediated antigen-specific T cell priming and expansion

**Fig 1:**
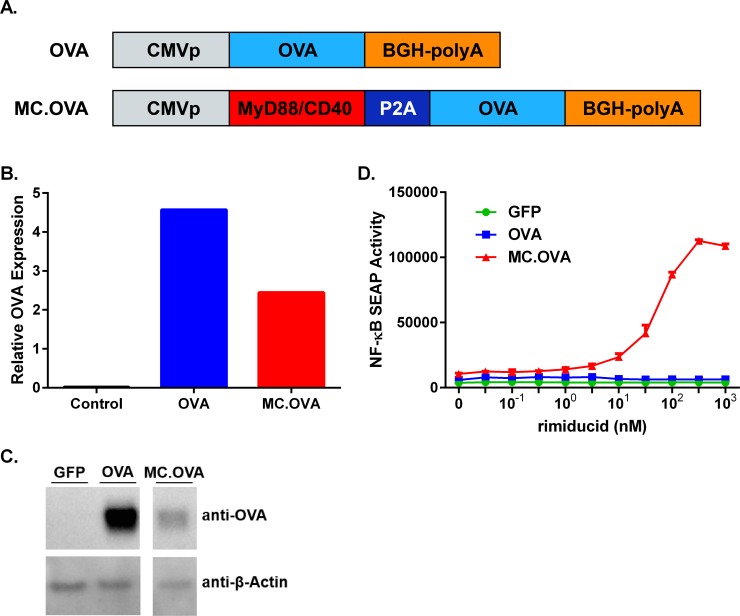
Design and Function of OVA and MC.OVA Vaccine Vectors. **(A)** Linear diagrams showing the functional features of the OVA (upper) and MC.OVA (lower) vaccine constructs. **(B, C)** 293 cells were transfected with 1 μg of the indicated plasmid. Lysates were probed for OVA expression by Western blot and expression was measured relative to β-actin. **(D)** 293 cells transfected with NF-κB SEAP reporter and either GFP, OVA, or MC.OVA expression plasmids. Cells were plated with increasing concentrations of rim.

Inclusion of immunological adjuvants has become standard practice to improve vaccine immunogenicity, including soluble agents, such as Freund’s incomplete adjuvant, aluminum salts, or soluble cytokines (e.g., IL-12, GM-CSF). Similarly, although *in vivo* electroporation (EP) inherently enhances several parameters of DNA vaccination, such as gene transfer, lymphocyte migration to draining lymph nodes, and T cell priming (**S1 Fig**) [17–20], inclusion of cytokine genes, such as GM-CSF or IL-12p70, within expression plasmids delivered by EP can further amplify vaccine efficacy [21,22]. In contrast to secreted cellular factors, use of intracellular signaling molecules as adjuvants for EP vaccines has not been well characterized. Since the membrane-localized MyD88/CD40 (MC) fusion protein is capable of propagating both TLR/IL1 Receptor-α and CD40 signaling pathways, we hypothesized that MC could also function as an effective adjuvant in EP-mediate DNA vaccines. Thus, we generated a vaccine vector, MC.OVA, containing MC in frame with ovalbumin, separated by a viral-derived 2A sequence (**Fig 1A**). Additionally, an ovalbumin-encoding vector (OVA) was generated as an antigen (Ag)-only control. Expression of OVA protein by Western blot was roughly similar in 293 cells transfected with OVA or MC.OVA, with MC.OVA expressing slightly lower levels, possibly due to the difference in plasmid size and transfection efficiency (**Fig 1B**). Previous signaling studies have indicated that rimiducid (rim)-mediated MC-oligomerization stimulates NF-κB transcriptional activity. Therefore, to confirm proper function of MC.OVA we used an NF-κB reporter assay. As expected, activation of NF-κB transcriptional activity in transiently transfected 293 cells was MC-dependent and induced by rim (**Fig 1C**).

**Fig 2:**
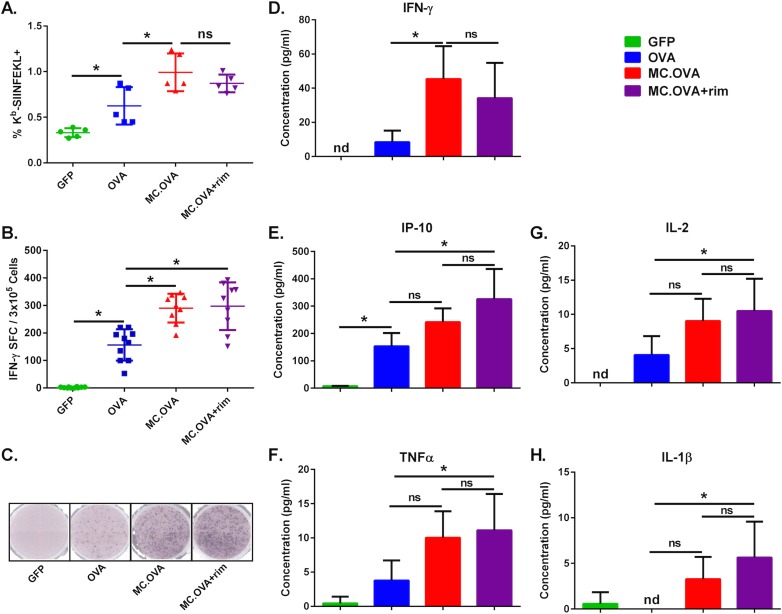
MC improves DNA EP vaccine immunogenicity compared to Ag alone. C57BL/6 mice were vaccinated with 25 μg of the indicated plasmids on days 0 and 21. One day following each vaccination, 1.25 mg/kg of rim was administered IP in the MC.OVA + rim treatment group. **(A)** On day 28 splenocytes were analyzed for numbers Ag-specific CD8^+^ T cells using H2-K^b^ SIINFEKL tetramers. Representative data from two independent experiments. **(B)** Ag-specific T cell function was assayed by IFN-γ ELISpot. Data from two independent experiments. **(C)** Representative images of ELISpot membranes. **(D-E)** Naïve mice were vaccinated on day 0 and again 20 days later with 25 μg of plasmid DNA followed by electroporation. MC.OVA + rim-treated mice were injected with 1.25 mg/kg IP the day following each vaccination. On day 27 splenocytes were isolated from the mice and incubated with SIINFEKL peptide ON and supernatants were collected and analyzed for 25 different cytokines/chemokines by multiplex (representative graphs shown). n = 5–10, *p<0.05, One-way ANOVA, Tukey correction for multiple comparisons, nd = not detectable.

To determine if MC could enhance the overall Ag-specific CTL response, we compared CTL activity in OVA *vs*. MC.OVA (± rim)-vaccinated mice. Flow cytometric analysis of OVA-specific T cell levels using H2-K^b^-based MHC tetramers loaded with immunodominant OVA peptide, SIINFEKL, revealed a significant increase in Ag-specific T cells in MC.OVA-treated mice compared to OVA vaccination alone; however, rim administration failed to further improve T cell responses compared to MC.OVA-vaccinated mice without rim, suggesting that the CMV immediate-early promoter-driven expression of vaccine-encoded MC provided sufficient basal activity from the adjuvant (**Fig 2A**). This was consistent with previously published data demonstrating increased MC basal activity when expressed as part of a 2A-containing cistron [11]. As a measure of OVA-specific T cell function, we also examined Ag-restimulated splenocytes for IFN-γ secretion *in vitro* by ELISpot (**Fig 2B and 2C**). Mice vaccinated with MC.OVA showed similar levels of spot-forming colonies (SFCs) independent of rimiducid administration, but significantly higher SFCs than OVA-vaccinated mice, supporting that MC, as designed, increases the overall number of functional Ag-specific T cells *in vivo*, in a rim-independent fashion.

### MC-APC primed T cells secrete higher levels of Tc1 cytokines

In our previous MC studies, we had determined that MC caused Tc1 skewing of CTL differentiation [10,11]. Therefore, we tested if this was the case in EP vaccination as well by examining cytokine secretion from Ag-stimulated splenocytes. As predicted, MC.OVA vaccination increased the quantity of IFN-γ secretion by OVA-specific cells (IFN-γ pg/ml / IFN-γ SFC) compared to mice vaccinated with OVA alone (**S2 Fig**). In addition, a broad multiplex analysis of SIINFEKL-stimulated splenocytes revealed that CD8^+^ T cells from MC.OVA-vaccinated mice secreted significantly higher levels of Tc1 cytokines compared to mice vaccinated with OVA alone. In particular, IFN-γ, TNFα, IL-2, IL-1β, and IP-10 secretion was significantly higher in MC.OVA-vaccinated mice compared to OVA-vaccination (**Fig 2D–2H**). Altogether, these data suggest that MC signaling increases the overall number and cytokine secretion of Tc1-polarized, Ag-specific CD8^+^ T cells.

### MC adjuvant increases anti-tumor response in tumor antigen-vaccinated mice

**Fig 3:**
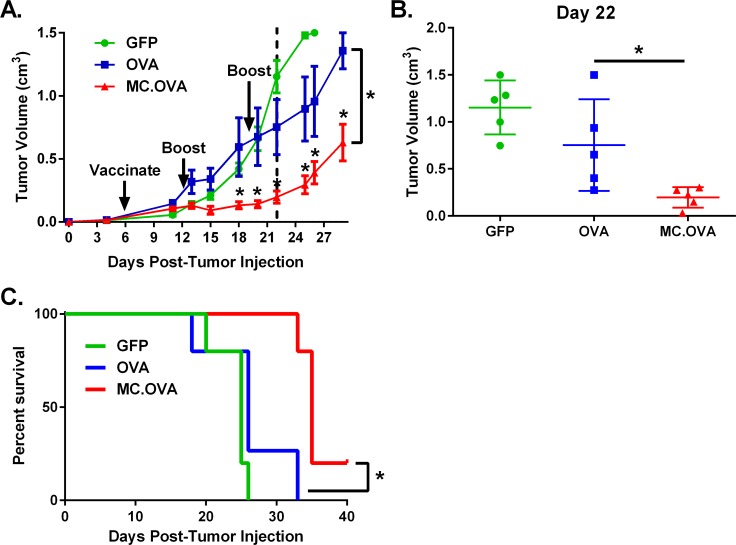
MC.OVA reduces tumor growth in B16-OVA bearing mice more effectively than vaccination with OVA alone. C57BL/6 mice were injected with 2 x 10^5^ B16-OVA cells subQ. Tumors were allowed to establish for 6 days before mice were randomized into treatment groups and vaccinated with 25 μg of either GFP, OVA, or MC.OVA plasmids on days 6, 12, and 19 post-tumor injection. Tumor volumes were determined by caliper measurements. **(A)** Tumor growth curves (error bars = SEM) for each treatment group. **(B)** Comparison of each group at day 22 post-tumor injection. Each point represents a single mouse (error bars SD). **(C)** Survival curves of tumor bearing mice. n = 5, *p<0.05, Two-way ANOVA, Holms-Šidák correction for multiple comparisons. Survival curve analysis by Log Rank (Mantel-Cox) test.

To better understand the improvement in anti-tumor effect of MC signaling over Ag vaccination alone, we compared growth of pre-established B16-OVA tumors in mice treated with either GFP, OVA, or MC.OVA. Mice were injected subQ with 2 x 10^5^ B16-OVA cells and tumors were allowed to establish before vaccinations were administered on days 6, 12, and 19 post-tumor injection. While OVA-vaccinated animals showed only a minor reduction in tumor growth, MC.OVA-vaccinated mice reflected a significant anti-tumor effect (**Fig 3A and 3B**). Interestingly, the divergence between OVA and MC.OVA groups occurred approximately 7 days after the initial vaccination and immediately following the first boost, consistent with a key role for adaptive immunity, which is delayed relative to innate immunity. Median survival was also increased in MC.OVA compared to OVA-treated mice (35 *vs*. 26 days post-tumor injection; **Fig 3C**). To confirm that the observed anti-tumor effect was not limited to this model, a similar tumor treatment experiment was performed in E.G7 tumor-bearing mice, yielding qualitatively similar results, in which MC.OVA outperformed OVA vaccination by delaying tumor growth (**S3 Fig**).

**Fig 4:**
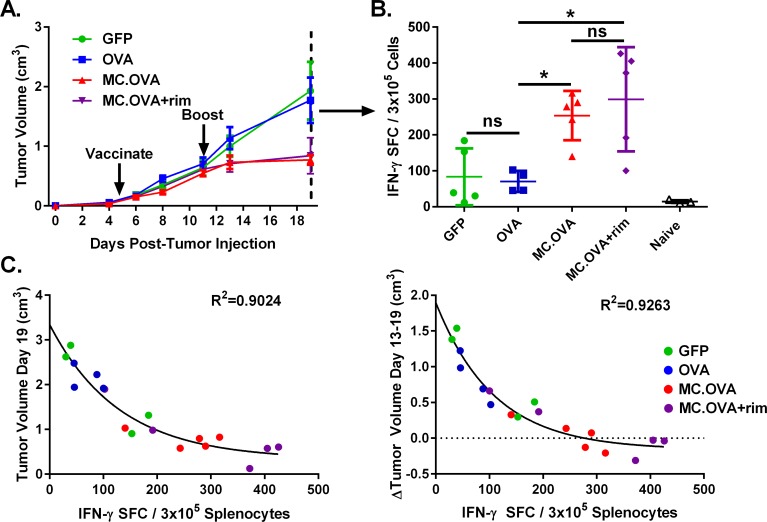
MC adjuvant improves TAg-specific CTL response in tumor-bearing mice. C57BL/6 mice were injected subQ with 5 x 10^5^ E.G7 tumor cells. Mice were vaccinated with 25 μg pDNA encoding either GFP, OVA, or MC.OVA on days 5 and 11 post-tumor injection. One day following vaccination, 1.25 mg/kg rim was administered IP in MC.OVA + rim-treated mice. **(A)** Tumor volumes were determined by caliper measurements. **(B)** The experiment was terminated on day 19 post-tumor injection and IFN-γ ELISpot was performed to quantify SIINFEKL-specific T cells. **(C)** Right panel: Correlation between day 19 tumor volume and IFN-γ SFC. Left Panel: correlation between change in tumor volume from day 13–19 vs IFN-γ SFC. R^2^ values determined by non-linear regression using a one phase decay curve-fit. n = 4–5, *p<0.05, One-way ANOVA, Tukey correction for multiple comparisons.

It is generally accepted that tumors can evade anti-tumor immunity through various mechanisms, including upregulation of immune-inhibitory receptors (e.g., PD-L1), immunosuppressive cytokines (e.g., TGF-β), and recruitment or induction of immunosuppressive cells (e.g., MDSCs) [23–25]. As such, it was possible that the modesty of the anti-tumor response observed in the OVA vaccine-treated mice was due to a functional deficit in tumor-localized T cells and not due to a reduction in the global number of circulating tumor-specific T cells. To explore this possibility, E.G7-bearing mice were vaccinated on days 5 and 11 post-tumor injection, and tumors allowed to grow until day 19 post-tumor injection (**Fig 4A)**, at which point SIINFEKL-specific IFN-γ secreting splenocytes were measured by ELISpot (**Fig 4B**). There were significantly higher levels of TAg-specific T cells in MC.OVA-vaccinated mice relative to OVA-vaccinated animals, consistent with increased anti-tumor immunity. Furthermore, there was a very high correlation between the level of splenic TAg-specific T cells and either tumor volume at the time of euthanasia or the change in tumor volume between booster vaccination and experiment termination (**Fig 4C**). This correlation between the level of TAg-specific T cells and the anti-tumor response is more consistent with an expanded anti-tumor response leading to a decrease in tumor growth *vs*. the tumor microenvironment having the key role in suppressing anti-tumor immunity in OVA-alone vaccination.

### MC adjuvant signals in major skin cell types

While, autologous *ex vivo* DC vaccines restrict Ag loading only to those DCs being manipulated, untargeted “off-the-shelf” DNA vaccines, such as one relying on *in vivo* EP, is not typically restricted in its transgene expression pattern, unless using tissue-specific promoters or RNA-based elements [26,27]. Therefore, unlike autologous *ex vivo* DC vaccines, EP vaccination results in Ag and adjuvant expression in many different cell types at the site of administration, including macrophages, DCs, and B cells, as well as other cell types, such as, keratinocytes (KCs), and fibroblasts (FBs) [26]. Therefore, it is possible that MC signaling and/or Ag expression or presentation by such atypical APCs could positively or negatively impact vaccine immunogenicity.

**Fig 5:**
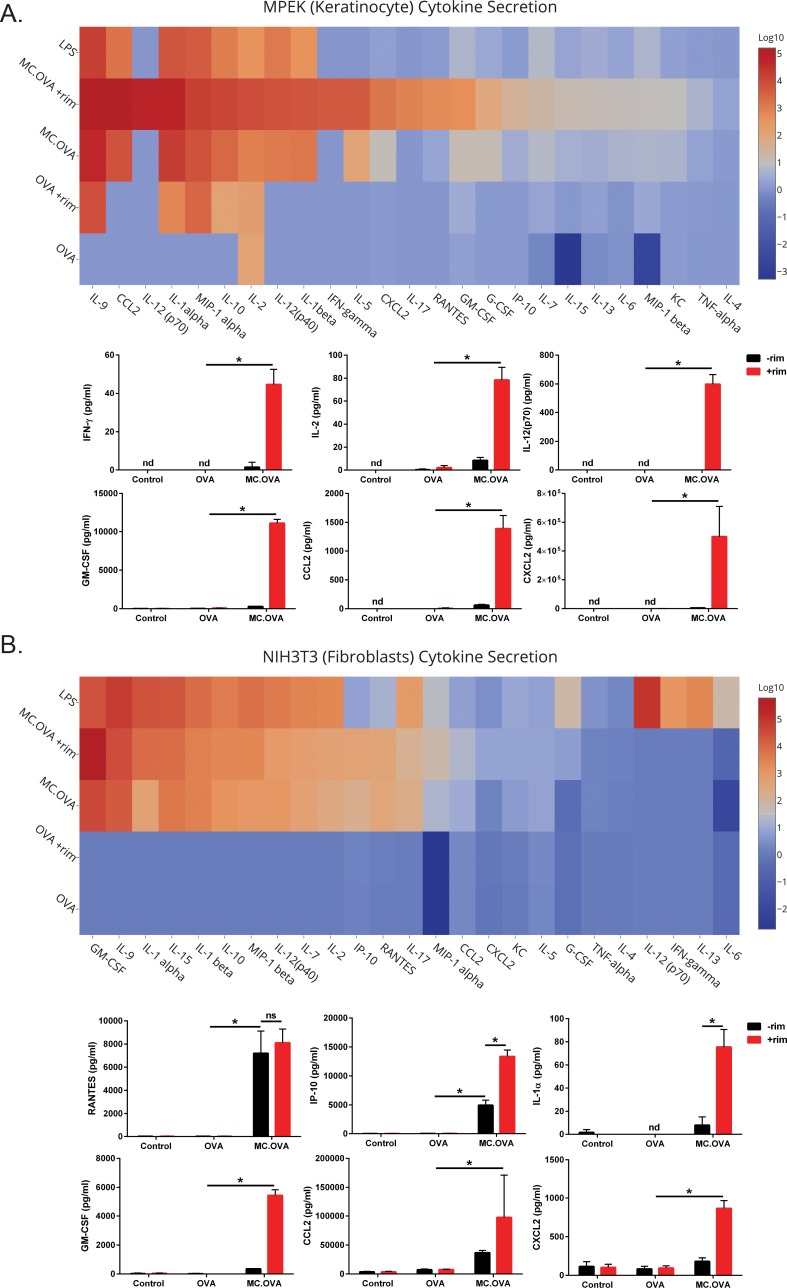
Cytokine secretion in keratinocytes and fibroblasts mediated by MC-activation. **(A-B)** B6-MPEK (MPEK), a keratinocyte cell line, and NIH3T3, a fibroblast cell line, were transduced with lentivirus encoding the GFP backbone alone (control cells), OVA, or MC.OVA. Polyclonal lines stably expressing these proteins were puromycin-selected to >90% transgene^+^ purity. 2 x 10^5^ cells were cultured with or without 10 nM rim for 48 hours. As a positive control, control cells were stimulated with 1 ng/ml LPS. Culture supernatants were analyzed for cytokine secretion by a 25-plex cytokine panel. **(A-B, Upper panels)** Fold induction of cytokines relative to control cell supernatants with 10 nM rimiducid. **(A-B, Bottom panels)** Representative graphs for each cell line showing the absolute quantitation of cytokines secreted in pg/ml. n = 3, *p<0.05, One-way ANOVA with Tukey correction for multiple comparisons, nd = not detectable.

To initially address the question of MC function in atypical APCs, we modified NIH3T3 fibroblast (FB) and MPEK keratinocyte (KC) cell lines to stably express copepod green fluorescent protein (copGFP) and either an empty backbone vector (negative control), OVA, or MC.OVA. Transgene positive cells were puromycin-selected and confirmed by flow cytometric analysis of copGFP expression. Secretion of inflammatory cytokines by FBs and KCs is an important function of their role in cutaneous immunity [28,29]. Therefore, cell culture supernatants were collected after 48 hours of incubation and cytokines were quantified by multiplex. This analysis revealed that MC-activated MPEK KCs and NIH3T3 FBs secreted a broad spectrum of inflammatory cytokines (**Fig 5**). When activated by MC, both cell types secreted extremely high levels of GM-CSF, CCL2, and CXCL2, all of which are important in the recruitment and/or differentiation of certain APC subsets. However, there was a distinct cytokine secretion profile between FBs and KCs. MC-activated MPEK KCs secreted high levels of IL-2, IFN-γ, and IL-12p70, all of which are important for cytotoxic T cell development and recruitment. In contrast, MC-activated NIH3T3 FBs secreted RANTES, IP-10, and IL-1α, which contribute to both innate and adaptive immunity.

**Fig 6:**
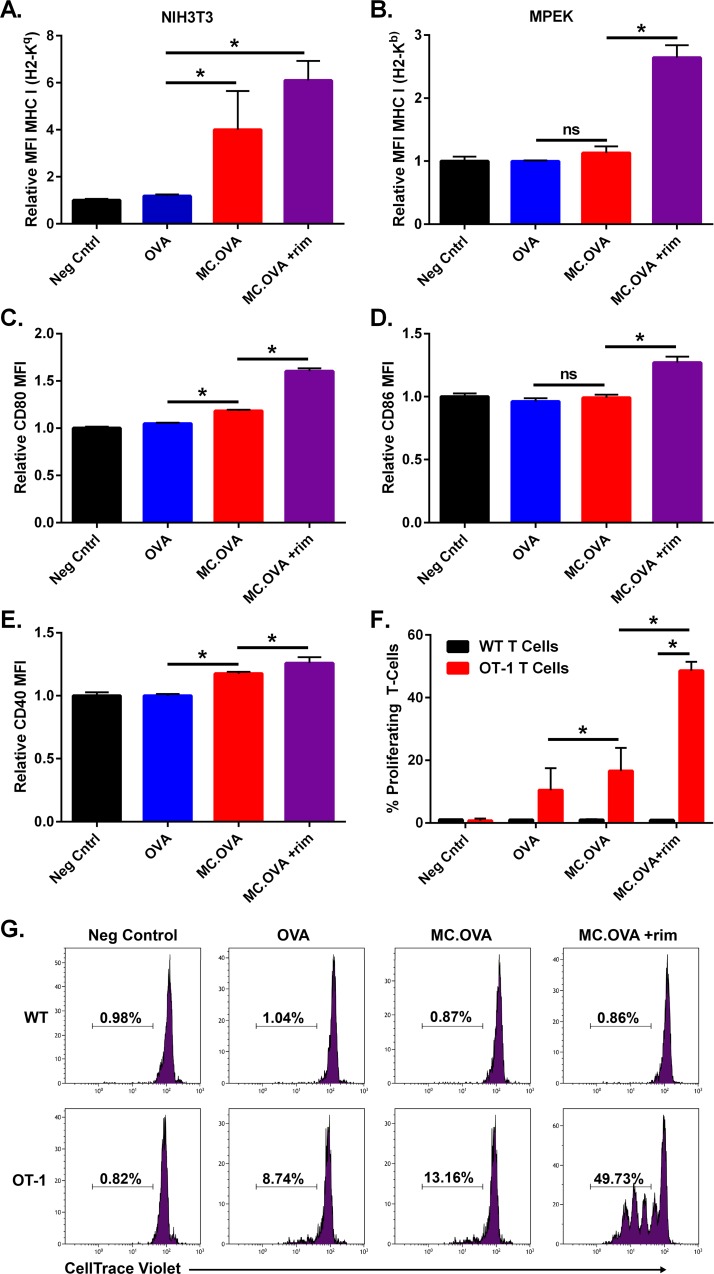
MC-mediated immune stimulatory properties of fibroblasts and keratinocytes. **(A)** NIH3T3 or **(B)** MPEK stable cells lines expressing either GFP alone (neg. control), OVA or MC.OVA were incubated for 60 hours. MC.OVA +rim had 10 nM rim present in the culture. After 60 hours, cells were harvested and MHC-I expression was analyzed by flow cytometry. **(C-E)** MPEK stable cells surface expression of CD80, CD86, and CD40. MFI values of live cells are reported relative to the mean MFI of negative control cells, transduced with an empty-vector, in each respective sample. **(F)** CD3^+^CD8^+^ OT-1 or WT T cells were purified by MACs and labeled with CellTrace violet (CTV) dye. Then 5 x 10^4^ CD3^+^CD8^+^ CTV dye-labeled OT-1 or WT T cells were incubated with 5 x 10^4^ B6-MPEK cells for 60 hours. By flow cytometry, live CTV^+^ cells were analyzed for CTV dye dilution as a measure of cell proliferation. **(G)** Representative histograms of live CTV^+^ cells. Percentages represent the percent of cells proliferating. n = 3, *p<0.05, One-way ANOVA, Tukey correction for multiple comparisons.

In addition to cytokine secretion, previous reports indicated that in response to pathogens and certain cytokines, KCs upregulate surface markers, such as MHC-I, MHC-II, and even B7.1 (CD80) [28]. Therefore, we wanted to know if MC was also able to upregulate MHC or costimulatory molecules on atypical APCs. Both NIH3T3 FBs and MPEK KCs upregulated MHC-I molecules on their surface when activated with MC (**Fig 6A and 6B**). Although not as dramatic as MHC-I, MC-activation of MPEK KCs resulted in modest, but significant, upregulation of co-stimulatory molecules CD80, CD86, and CD40 (**Fig 6C–6E**). Interestingly, MC-activated FBs also proliferated at an increased rate *in vitro* compared to parental and control cells (**S4 Fig**). Overall, MC enhanced the “immune-like” phenotype of both NIH3T3 FBs and MPEK KCs in terms of cytokine secretion and cell surface marker expression.

### MC-activated keratinocytes can prime antigen-specific T cell expansion *in vitro*

Curiously, MC-modified KCs appeared to reflect many of the hallmarks of professional APCs required for naïve T cell priming. Similarly, previous work showed that IFN-γ stimulated keratinocytes can process and present peptide to both CD4^+^ and CD8^+^ T cells [30]. Therefore, we wanted to determine if MC-MPEK KCs could directly prime and expand naïve, Ag-specific T cells *in vitro*. The use of OT-1 T cells, which are transgenic for an H2-K^b^-SIINFEKL-reactive αβ TCR, permit the interrogation of T cell stimulation in the context of a well-defined and Ag-restricted T cell population. Therefore, to test this hypothesis, KCs were co-cultured at a 1:1 ratio with CellTrace violet (CTV) dye-labeled, CD3^+^CD8^+^ OT-1 T cells. T cell proliferation was measured by flow cytometry analysis of dye dilution, a result of cell division. Interestingly, while there were low levels of OT-1 proliferation in OVA-MPEK co-cultures, rim-activated MC-MPEK cells induced significantly higher levels of OT-1 proliferation (49.73% vs 8.74%) (**Fig 6F–6G**). The requirement of MC for the MPEK KCs to induce high levels of T cell proliferation suggests that either inflammatory cytokines, surface costimulation, high surface MHC-I, or a combination of these is required for effective, KC-mediated direct T cell priming.

### MC adjuvant functions in atypical APCs to enhance vaccine-mediated CD8^+^ T cell responses

As MC signaling appeared to impart a proinflammatory phenotype onto atypical APCs *in vitro*, we wanted to determine if this observation extended *in vivo*. The microRNA (miRNA), miR142-3p, has been previously described as being expressed exclusively by cell types differentiated from the hematopoietic lineage, and is therefore not expressed in other cell types, such as, KCs, skeletal muscle, or epithelial cells present at the vaccination site [13]. Based on previous reports, inclusion of miR142-3p target sequence into the 3’ UTR of an expression vector results in degradation of the vector-encoded mRNA transcript in hematopoietic cells, mediated by RISC complexed miR142-3p, preventing protein expression [31,32]. Therefore, we speculated that incorporating miR142-3p target sequence into the 3’ UTR of our vaccine vector could help distinguish whether the putative role of MC stimulation in atypical APCs *in vivo* was sufficient for adjuvant-enhanced vaccine efficacy or if additional direct MC-function in hematopoietic cells is required. To accomplish this, a tandem tetramer of the targeting sequence for miR142-3p (miR142T) was inserted in the 3’ UTR of the vaccine backbone and validated for hematopoietic cell-specific repression of transgene Ag expression and MC-function by miR142T (**S5 Fig**). Subsequently, OVA and MC.OVA-encoding cassettes were cloned into the validated miR142T backbone to generate OVA.miR142T and MC.OVA.miR142T vaccine vectors, respectively.

**Fig 7:**
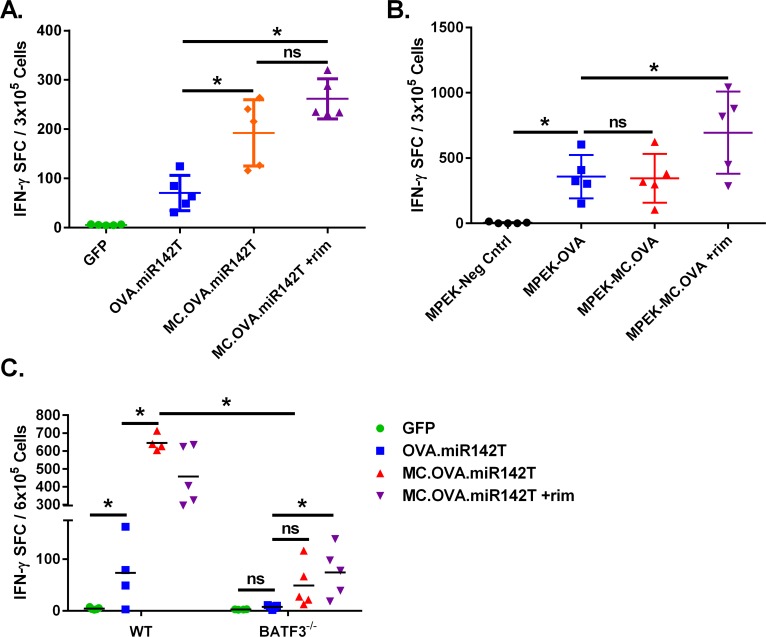
Expression of MC and Ag in cutaneous atypical APCs contribute to adjuvant-enhanced EP vaccine-mediate Ag-specific CD8^+^ T cell priming and is partly CD8α^+^/CD103^+^ DC-independent. C57BL/6 mice were vaccinated on days 0 and 21 with 25 μg pDNA by EP, rim was administered 1.25 mg/kg IP the day following each vaccination in MC.OVA.miR142T + rim-treated mice. **(A)** Splenocytes were extracted 7 days after the final vaccination (day 28) and restimulated with SIINFEKL peptide overnight. IFN-γ SFCs were quantitated by ELISpot. n = 5, *p<0.05, One-way ANOVA, Tukey correction for multiple comparisons. **(B)** C57BL/6 mice were injected in alternating flanks 21 days apart with 1 x 10^5^ MPEK cells stables (neg. control, OVA, MC.OVA). Rimiducid was administered at 1.25 mg/kg IP 24 hours after each injection of cells. 7 days following the second injection, splenocytes were analyzed for SIINFEKL-specific IFN-γ-secreting CD8^+^ T cell by ELISpot. Error bars represent 95% confidence interval. *p<0.05 when compared to MPEK-OVA, One-way ANOVA, Dunnett’s correction for multiple comparisons. **(C)** Either C57BL/6 or *Batf3*^-/-^ mice were vaccinated in the same manner as described for panel A. SIINFEKL-specific IFN-γ-secreting splenocytes were quantitated by ELISpot. n = 4–5, *p<0.05, One-way ANOVA, Tukey correction for multiple comparisons within mouse strain.

*In vitro*, MC activation was able to induce a very strong proinflammatory phenotype in KCs and FBs, unlike Ag expression alone. Therefore, we hypothesized that *in vivo*, limiting MC activation and Ag-expression to atypical APCs with miR142T would still result in an MC-mediated boost in vaccine immunogenicity. To test this, naïve mice were vaccinated twice, 21 days apart, with either GFP, OVA.miR142T, or MC.OVA.miR142T. Splenocytes from immunized animals were isolated 6 days following the last vaccination, and levels of Ag-specific CD8^+^ T cells were quantitated by IFN-γ ELISpot with SIINFEKL peptide restimulation (**Fig 7A**). As hypothesized, despite miR142-3p-restricted expression, MC was able to enhance immune responses, as MC.OVA.miR142T-vaccinated mice displayed higher numbers of IFN-γ-secreting CD8^+^ T cells relative to OVA.miR142T-vaccinated mice. These results indicate that MC is able to enhance the priming of naïve Ag-specific T cells independent of direct signaling in immune cells.

To show that MC-activated, atypical APCs are able to act directly as a vaccine-carrier, we utilized gene-modified MPEK KCs, which are syngeneic to C57BL/6 mice, as an alternative vaccine modality. Vaccination with either OVA-MPEK or MC.OVA-MPEK without rimiducid primed similar levels of SIINFEKL-specific IFN-γ-secreting CD8^+^ T cells; however, rimiducid-activated MC.OVA-MPEK vaccination enhanced the SIINFEKL-specific CD8^+^ T cell response, supporting that *in vivo* MC function in atypical APCs contributes to adjuvant-enhanced immunogenicity (**Fig 7B**). The requirement for rim in this model, is potentially the result of lower transgene expression levels in stably integrated cell lines, compared to high transient expression resulting from electroporated DNA.

Taken together, these data indicate that while Ag expression alone in atypical APCs generates a suboptimal vaccine response, addition of MC signaling is able to augment the Ag-specific T cell response through direct activity in local cutaneous cell types such as, keratinocytes.

### Improved vaccine efficacy via MC signaling and antigen expression in atypical APCs is partially independent of BATF3-dependent cross-priming DCs

To determine if MC adjuvant function in atypical APCs was dependent on professional APCs for enhancing Ag-specific T cells responses, we vaccinated mice deficient in the *Baft3* transcription factor [33]. Mice deficient in *Batf3* lack CD8α^+^/CD103^+^ DC subsets, which are responsible for the cross-presentation of Ags from the skin, and are unable to generate CD8^+^ T cell responses to DNA vaccine encoded Ags [34]. Therefore, naive *Batf3*^-/-^ or WT controls were vaccinated by EP (**Fig 7C**). As before, WT mice vaccinated with MC.OVA.miR142T either with or without rim demonstrated an enhanced Ag-specific CD8^+^ T cell response as compared to animals vaccinated with OVA.miR142T alone. Although all WT mice vaccinated with OVA-expressing vector exhibited SIINFEKL-specific CD8^+^ T cell responses, only *Batf3*^-/-^ mice vaccinated with MC.OVA.miR142T ±rim were able to generate a significant increase in Ag-specific CD8^+^ T cells over background. However, the observed Ag-specific T cell response to MC.OVA.miR142T in *Batf3*^-/-^ was significantly reduced as compared to similarly treated WT mice (≈15% of WT levels). These data suggest, that while the CD8α^+^/CD103^+^ dendritic cell subset is necessary for the full magnitude of MC-enhance vaccine responses, the observed MC effect does not wholly require and is partially independent of BATF3-dependent DCs.

### Antigen expression and MC signaling in atypical APCs improves T cell cytotoxicity and anti-tumor response

**Fig 8:**
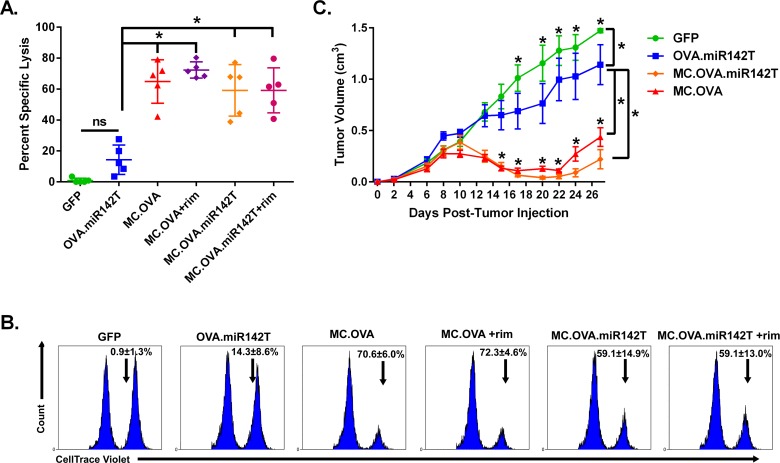
Anti-tumor and cytotoxic CD8^+^ T cell responses are increased by cutaneous atypical APCs expressing MC and T-Ag. **(A-B)** *In vivo* CTL assay. Splenocytes from naïve, syngeneic C57BL/6 mice were isolated and labeled with either 0.5 μM (Lo) or 5 μM (Hi) CTV dye, then pulsed with 10 ng of either irrelevant H2-K^b^ ICPMYARV (β-gal) peptide (Lo), or target H2-K^b^ SIINFEKL (OVA) peptide (Hi). Ag-pulsed and dye-labeled splenocytes were mixed 1:1 (Hi:Lo) and a total of 1e7 cells per mouse was injected IV into vaccinated mice 7 days after the last vaccination. After 7 hours, splenocytes were extracted and analyzed for the presence of CTV dye-labeled cells. The Hi:Lo CTV^+^ cell ratio was proportional to the levels of target-specific killing. **(B)** Representative histograms of live, CTV^+^ splenocytes. Values are the mean ± SD of % specific lysis of target cells. n = 4–5, *p<0.05, One-way ANOVA, Tukey correction for multiple comparisons. **(C)** C57BL/6 mice were injected subQ with 1e6 E.G7 tumor cells on day 0. Tumors were allowed to establish for 2 days. On days 2 and 9, mice were vaccinated with 25 μg pDNA by EP. Tumor volumes were determined by caliper measurements. n = 10, *p<0.05 when compared to OVA.miR142T, Two-way ANOVA with repeated measures, Holm-Šidák correction for multiple comparisons, error bars represent SEM.

To evaluate whether Ag-specific CD8^+^ T cells in miR142T vector-vaccinated mice were functionally primed to kill target cells we assayed the *in vivo* ability of vaccine-primed CTLs to eliminate peptide-loaded target cells. To test the cytotoxic potential of these CD8^+^ T cells, animals were vaccinated as before, but this time, animals were injected IV with SIINFEKL-pulsed splenocytes from naïve syngeneic mice 7 days following the last vaccination. After 7 hours, splenocytes from vaccinated mice were isolated and analyzed for specific lysis of adoptively transferred target cells (**Fig 8A and 8B**). Importantly, MC.OVA.miR142T ± rim vaccination generated an Ag-specific T cell response capable of significantly higher specific lysis compared to OVA.miR142T and similar to that of MC.OVA vaccination. Tetramer analysis for SIINFEKL-specific CD8^+^ T cells indicated that the levels of total Ag-specific T cells in MC.OVA.miR142T-vaccinated mice corresponded to the level of target-specific lysis (**S6 Fig**).

Finally, to assess the influence of MC signaling in atypical APCs on the therapeutic efficacy of EP vaccination, we evaluated this treatment in E.G7 tumor-bearing mice (**Fig 8C**). Tumors were established subQ prior to the first vaccination with plasmids expressing either GFP (negative control), OVA.miR142T, MC.OVA.miR142T, or MC.OVA. Animals received a booster 7 days after the first vaccination (day 9 post-tumor injection). Vaccination with MC.OVA encoding constructs, regardless of miR142T inclusion, was able to significantly reduce tumor volume compared to vaccination with OVA.miR142T alone. These data support that MC-enhanced EP-based vaccination does not depend exclusively on direct Ag/adjuvant expression in APCs, but can contribute to DNA vaccine immunogenicity *in situ* via a variety of cell types present. This mechanism helps to explain the simplicity and observed effectiveness of DNA vaccination, particularly intradermal administration, where only a small fraction of electroporated cells are likely to be APCs.

## Discussion

DNA vaccines continue to draw broad interest in clinical applications for both infectious and malignant disease, as well as their intersection, where viral oncogenes or chronic inflammation can contribute to certain cancers. Although most trials have established good safety and tolerability in patients, clinical data from DNA vaccine trials have been met with mixed reaction, since efficacy has been underwhelming, illustrating the need for improved immunogenicity [3,4].

Many strategies to augment the efficacy of therapeutic cancer vaccines have been and are currently being clinically evaluated [35,36], such as (i) vaccination with a tumor-associated Ag-overexpressing heat-killed strain of *Saccharomyces cerevisiae* (NCT01519817) [2], (ii) PROSTVAC, a prime-boost vaccine using vaccinia and fowlpox viral vectors encoding prostate-specific antigen (PSA) along with 3 immunostimulatory molecules (i.e., CD80, CD58, and ICAM-1) in metastatic prostate cancer patients (NCT01322490) [37,38], and (iii) GM-CSF and IL-12p70-encoding plasmids being used in Phase I/II clinical trials for various tumor indications [39,40]. Interestingly, use of intracellular signaling molecules, like MyD88 or the cytoplasmic domains of pro-inflammatory TNFR family members (e.g., CD40), as adjuvants has been largely overlooked. Our prior proof-of-concept studies and implementation of MC adjuvant in *ex vivo* DC and adenovirus-mediated vaccines, have shown that the use of molecules such as these, which lie “upstream” in pro-inflammatory signaling cascades, have the potential advantage of inducing a pleiotropic immunostimulatory phenotype, which is, in turn, capable of generating many costimulatory factors or cytokines, thus amplifying adjuvant potency [10,11].

Herein, we demonstrate that MC incorporation into “off-the-shelf” EP vaccines significantly enhanced vaccine efficacy via Ag-specific CD8^+^ CTL induction and expansion. We also show that MC signaling enhanced anti-tumor responses in mice, correlating with expansion of Ag-specific CTLs, and that MC-TAg-expanded CD8^+^ CTLs secreted higher levels of Tc1 cytokines. These data are in line with previous work using MC as an adjuvant in cancer vaccines [10,11]. The quantitative and functional immune data suggest that improved anti-tumor efficacy is directly correlated with an increase in TAg-specific CD8^+^ T cells generated by vaccination. Our earlier studies of MC function in dendritic cells demonstrated that MC increases the magnitude of T cell responses through increased Tc1-polarizing cytokine secretion, costimulatory marker expression, and migration to draining lymph nodes. Additionally, although MC was designed to contain rimiducid-binding domains (i.e., FKBP12_v36_), rimiducid-dependent MC oligomerization was generally not essential in this application. Likely explanations for this observation are that sufficient basal MC activity can result from protein overexpression, resulting in a higher frequency of stochastic MC aggregation, or alternatively, inefficient 2A-mediated peptide separation can lead to “stickier” MC-Ag fusions that may further contribute to spontaneous MC aggregation. Furthermore, the “threshold” of MC signaling may vary by cell type, due to variations in the steady-state prevalence of downstream signaling molecules. We are currently working to better understand how transgene design elements affect MC signaling properties. Serendipitously, however, the loss of rimiducid-dependence in this context may be advantageous, as it reduces the complexity of this vaccine approach without creating a safety concern, due to the transience of non-integrating DNA-based vaccines.

While most MC studies, as well as those of other genetic adjuvants, have focused on the effects on professional APCs, we sought to better understand MC signaling in the more numerous atypical APC types found *in situ*. Restricting Ag expression to atypical APCs by miR142-3p generated suboptimal immunogenicity; however, addition of miR142-3p-restricted MC signaling produced TAg-specific CD8^+^ T cell responses comparable to those resulting from unrestricted MC expression, which were sufficient for tumor control. This suggests that MC-enhanced vaccine efficacy is largely the result of adjuvant function in atypical APCs, either by directly or indirectly modulating the downstream immune response.

As front-line immune sentinels of the skin, KCs express pathogen recognition receptors (e.g., TLRs), antimicrobial peptides, and assemble activated inflammasomes, quickly sensing and responding to pathogenic insult [41]. Additionally, KCs have been implicated in immune-mediated inflammatory pathologies of the skin. For example, KCs can drive skin auto-reactivity via Ag presentation to CD4^+^ and CD8^+^ T cells in animal models of psoriasis and toxic epidermal necrolysis [41–43]. Therefore, KCs are central to the cutaneous immune system, capable of driving immune homeostasis or dysfunction. As our data indicate, MC is capable of dramatically altering the immune phenotype of KCs *in vitro*. MC-enabled KCs exhibited hallmark APC attributes for CD8^+^ T cell priming: upregulation of MHC-I (Signal 1) and costimulatory molecules (Signal 2), and robust secretion of Tc1 cytokines/chemokines (Signal 3). Furthermore, MC activation of KCs permitted direct *in vitro* expansion of Ag-specific T cells in the absence of professional APCs, indicating that MC-enhanced KCs are able to effectively process and present antigenic peptides on MHC-I complexes. One potential mechanism may be mediated by IFN-γ-induced Ag uptake, processing, and presentation by KCs [30]; however, other KC-derived factors, including T cell stimulating cytokines, like IL-2 or IL12p70, may also be involved. Altogether, these data illustrate the ability of MC to enhance the naïve T-cell stimulatory functions of cutaneous atypical APCs.

Although, atypical APCs display an augmented ability to directly stimulate naïve Ag-specific T cells *in vitro*, our data supports that the stimulatory effect of MC expression in atypical APCs *in vivo* partly results from modulation of cutaneous professional APCs. For example, MyD88-deficient keratinocytes impair Langerhans cell emigration from the skin in a model of atopic dermatitis, suggesting that MC-mediated signaling downstream of MyD88 may permit efficient migration of dermal-resident DCs to draining lymph nodes due to keratinocyte-derived factors [44]. Moreover, *in vitro* MC signaling in keratinocytes induced secretion of GM-CSF and CCL2, which can contribute to recruitment and activation of APCs [45–47]. In particular, GM-CSF has been shown to play an important role in the recruitment and function of CD8α^+^/CD103^+^ DCs, which are required for maximum MC-enhanced T cell responses [48–50].

Prior reports underlie the importance of CD8α^+^/CD103^+^ DCs in cross-priming naïve T cells, as mice that are deficient in the transcription factor, BATF3, specifically lack the CD8α^+^/CD103^+^ DC subset and are likewise unable to prime CD8^+^ T cell responses following vaccination with MHC-I/peptide single-chain trimers [34]. In addition to normal Ag-uptake during cross-priming of MHC class I peptides, CD8α^+^/CD103^+^ DCs can rely on “cross-dressing” to prime naïve CD8^+^ T cells, whereby peptide:MHC complexes are transferred from a bystander cell to the surface of a professional APC. We observed that MC signaling upregulated MHC-I expression on KCs and FBs *in vitro*, which could increase CD8α^+^/CD103^+^ DCs cross-dressing *in vivo* [33,51]. In the absence of *Batf*3-dependent conventional DCs, MC adjuvant was still able to elicit significant numbers of Ag-specific CD8^+^ T cells, although at levels substantially reduced compared to WT mice. During certain intracellular infections, such as *T*. *gondii*, family members *Batf* and *Batf2* are able to compensate for *Batf3* in CD8α^+^/CD103^+^ DCs development, which could be mimicked by MC signaling in atypical APCs and should be further investigated [52]. Nonetheless, these data suggest that while MC adjuvant-enhanced vaccine efficacy is largely achieved through increased cross-priming via CD8α^+^/CD103^+^ DCs, MC-signaling in atypical APCs also supports an alternative albeit weaker mechanism of Ag-presentation and T-cell priming, which is independent of BATF3-dependent DC cross-presentation. These alternative mechanisms could include recruitment and Ag and/or peptide:MHC transfer to other less potent DC subsets, activated macrophages, or even B cells, possibilities that will be investigated in future studies.

While the use of intracellular signaling molecule adjuvants, such as MC, have distinct advantages, by contributing to pleiotropic immune stimulation, they also present novel considerations in their design and utility. MC expression in “bystander,” atypical APCs appears to improve vaccine efficacy without any obvious toxicities; nevertheless, the use of broadly active signaling molecules, such as MyD88, will likely require additional monitoring for adverse effects following non-targeted gene expression. For example, MyD88 is required for RAS-mediated transformation and carcinogenesis in keratinocytes, and therefore, despite transient expression, repeated MC administrations to the skin should be closely observed. However, MC-based adjuvant vaccines that use improved delivery methods and appropriate Ag selection (possibly leveraging recent breakthroughs in neo-antigen discovery [53]), in combination with other immunostimulatory strategies, such as checkpoint blockade antibodies (e.g., ipilimumab or pembrolizumab), could provide an effective treatment modality for some cancers.

Altogether, this study provides novel insights into the functional significance of manipulating intracellular signaling proteins as adjuvants for DNA vaccination, and to our knowledge is the first report to describe the action of a genetic adjuvant in atypical APCs to augment the efficacy of a therapeutic DNA vaccine against solid tumors. Importantly, our results have a direct impact on the guidance of future DNA vaccine strategies, illustrating the significance of adjuvants that not only promote relevant activation of standard immune cell subsets, but also atypical APCs, which can greatly influence the therapeutic outcomes.

## Supporting Information

S1 Fig In Vivo Electroporation enhances DNA Vaccination. **(A)** Mice injected subQ with FFLuc reporter plasmid with (left side) or without (right side) EP. **Left Panel:** Representative images of FFLuc activity on days 1, 5, and 8 post-reporter injection. **Right Panel:** Average radiance of EP- or non-EP-treated limbs day 5 post-treatment. Approximately 1500-fold greater total signal observed than in limbs receiving FFLuc plasmid without EP. n = 3–6. **(B) Left Panel:** no treatment (NT), PBS injection + EP, 25 μg plasmid DNA (pDNA) alone, or 25 μg pDNA + EP. 24 hours later, cells in DLNs were enumerated. Mice treated with either pDNA or pDNA + EP, but not PBS + EP (no pDNA) showed marked increases in LN cells, suggesting that pDNA was the primary inflammatory mediator. **Right Panel**: In a similar experiment, the percentage of CD19^+^, CD3^+^, and CD11c^+^ cells within DLNs was measured by flow cytometry. The absolute number of all interrogated leukocyte subsets (CD3^+^, CD19^+^, and CD11c^+^) increased in EP + pDNA-treated mice, and their relative composition changed was also altered by EP + pDNA. The reduction in the relative number of T cells (51.1% *vs*. 31.1% CD3^+^) was compensated by an increase in the relative number of B cells (30.6% *vs*. 47.2% CD19^+^), while CD11c^+^ cells stayed approximately unchanged (18.4% *vs*. 21.7%) **(C)** C57BL/6 mice were vaccinated with 50 μg GFP or LacZ with or without EP. After 7 days, splenocytes were restimulated with relevant β-gal_497-504_ (ICPMYARV) H2-K^b^-restrictued peptide, and Ag-specific T cell responses were measured by IFNγ ELISpot. n = 5 *p<0.05, (A) unpaired student’s t-test (B-C) One-way ANOVA with Tukey correction for multiple comparisons.
(TIF)

S2 Fig MC adjuvant improves OVA specific immune response in mice. Naïve mice were vaccinated on day 0 and again 20 days later with 25 μg of plasmid DNA followed by electroporation. Some mice were injected with 1.25 mg/kg IP the day following each vaccination. On day 27 splenocytes were isolated from the mice and analyzed for Ag-specific T cells by **(A)** IFN-γ ELISpot and levels of IFN-γ secretion from SIINFEKL stimulated splenocytes, were quantified by ELISA. **(B)** ELISA-measured IFN-γ pg/ml was divided by SFC values for each replicate and the values plotted to give an estimate of the amount of IFN-γ secreted by each SIINFEKL-specific T cell. Values for MC.OVA ± rim were pooled. Analysis by One-Way ANOVA with Tukey correction for multiple comparisons, n = 5–10, *p<0.05
(TIF)

S3 Fig MC improves anti-tumor efficacy of *in vivo* electroporation vaccine against E.G7 tumors *in vivo*. Mice were injected subQ with 1 x 10^6^ E.G7 cells on day 0. Mice were randomized on day 4 to normalize inter-group tumor volume. On days 5, 11, and 21 mice were vaccinated with 25 μg of the indicated plasmid in alternating flanks by EP. One day following each vaccination, 1.25 mg/kg rim was administered in MC.OVA + rim-treated mice. Tumor volumes were measured using calipers and the following equation; Volume (cm^3) = (0.5236) x L x W^2. Dotted lines in left hand panel indicate the time point at which groups were compared in the right panel. n = 4–5, *p<0.05, Analysis by One-Way ANOVA with Tukey correction for multiple comparisons.
(TIF)

S4 Fig FBs proliferate at an accelerated rate *in vitro* when activated by MC. 2.5 x 10^5^ of either negative control or MC-modified NIH3T3 FBs were plated into a 96-well flat-bottom plate. The wells were then imaged at 6 hour intervals using an IncuCyte live cell analysis system. Images were analyzed for percent confluency of bright field well-images. n = 6, *p<0.05 compared to Neg Control +rim, Two-way ANOVA with repeated measures and Tukey correction for multiple comparisons.
(TIF)

S5 Fig miRNA targeting sequence miR142T inhibits expression of vaccine in hematopoietic lineage cell types. **(A)** Non-hematopoietic HEK-293 or hematopoietic IC21 cells were cotransfected with NF-κB SEAP reporter and either GFP, MC.Antigen (MC.PSMA), or MC.Antigen.miR142T (MC.PSMA.miR142T). Transfected cells were plated with dilutions of rimiducid. SEAP activity was assayed after 24 hours. **(B)** Non-hematopoietic HEK-293 cells were transfected or hematopoietic EL4 cells were nucleofected with a plasmid expressing either Antigen (PSMA, Left panel) or MC.Antigen (MC.PSMA, Right panel) with or without the miR142T sequence. After 24 hours Ag (PSMA) expression was assessed by flow cytometry. Values relative to corresponding -miR142T vector transfected cells. **(C)** Top Panel: EP of parental vectors results in global expression of transgene in all cell types at the site of administration, including APCs, as indicated by the green. Bottom Panel: EP of vaccine vectors containing miR142T miRNA target sequence prevent expression of vaccine-encoded proteins in cells differentiated from a hematopoietic lineage (e.g., DCs and macrophages), however expression in other cells types (e.g., keratinocytes) is still permitted.
(TIF)

S6 Fig H2-K^b^-SIINFEKL Tetramer analysis of EP Vaccinated mice. C57BL/6 mice were vaccinated on days 0 and 21 with 25 μg pDNA by EP. Some mice received rim, administered 1.25 mg/kg IP, the day following each vaccination. On day 28, 7 hours prior to termination, syngeneic splenocytes were adoptively transferred into mice for an *in vivo* CTL assay (Fig 8A and 8B). **(A)** Splenocytes were extracted 7 days after the final vaccination (day 28) and analyzed for H2-K^b^-SIINFEKL Tetramer^+^ CD3^+^CD8^+^ T cells. **(B)** Gating strategy to remove adoptively transferred splenocytes by CTV. **(C)** Representative scatter plots for each group. Percentages are mean values ± SD. n = 5, *p<0.05, One-Way ANOVA with Holm-Šidák correction for multiple comparisons to OVA.
(TIF)

S1 Supplemental Methods Materials and methods for supplemental figures. (DOCX)
