# Supplementary figures and images for: MyD88/CD40 Genetic Adjuvant Function in Cutaneous Atypical Antigen-Presenting Cells Contributes to DNA Vaccine Immunogenicity
Source: PLoS One. 2016 Oct 14;11(10):e0164547. doi: 10.1371/journal.pone.0164547 (PMC5065236; doi:10.1371/journal.pone.0164547)

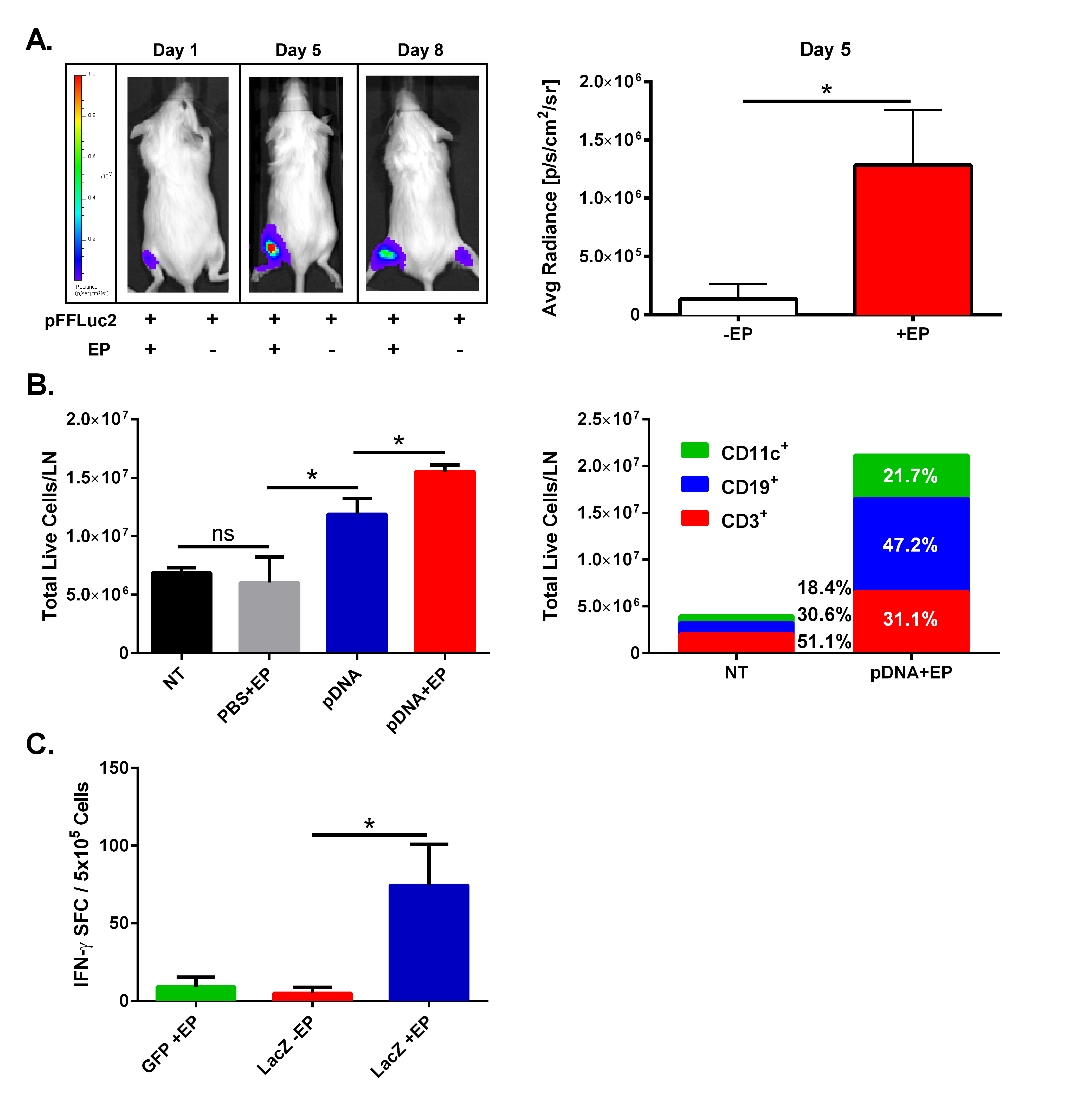

Supplement: S1 Fig — (A) Mice injected subQ with FFLuc reporter plasmid with (left side) or without (right side) EP. Left Panel: Representative images of FFLuc activity on days 1, 5, and 8 post-reporter injection. Right Panel: Average radiance of EP- or non-EP-treated limbs day 5 post-treatment. Approximately 1500-fold greater total signal observed than in limbs receiving FFLuc plasmid without EP. n = 3–6. (B) Left Panel: no treatment (NT), PBS injection + EP, 25 μg plasmid DNA (pDNA) alone, or 25 μg pDNA + EP. 24 hours later, cells in DLNs were enumerated. Mice treated with either pDNA or pDNA + EP, but not PBS + EP (no pDNA) showed marked increases in LN cells, suggesting that pDNA was the primary inflammatory mediator. Right Panel: In a similar experiment, the percentage of CD19+, CD3+, and CD11c+ cells within DLNs was measured by flow cytometry. The absolute number of all interrogated leukocyte subsets (CD3+, CD19+, and CD11c+) increased in EP + pDNA-treated mice, and their relative composition changed was also altered by EP + pDNA. The reduction in the relative number of T cells (51.1% vs. 31.1% CD3+) was compensated by an increase in the relative number of B cells (30.6% vs. 47.2% CD19+), while CD11c+ cells stayed approximately unchanged (18.4% vs. 21.7%) (C) C57BL/6 mice were vaccinated with 50 μg GFP or LacZ with or without EP. After 7 days, splenocytes were restimulated with relevant β-gal497-504 (ICPMYARV) H2-Kb-restrictued peptide, and Ag-specific T cell responses were measured by IFNγ ELISpot. n = 5 *p<0.05, (A) unpaired student’s t-test (B-C) One-way ANOVA with Tukey correction for multiple comparisons. (TIF) [file pone.0164547.s001.tif]

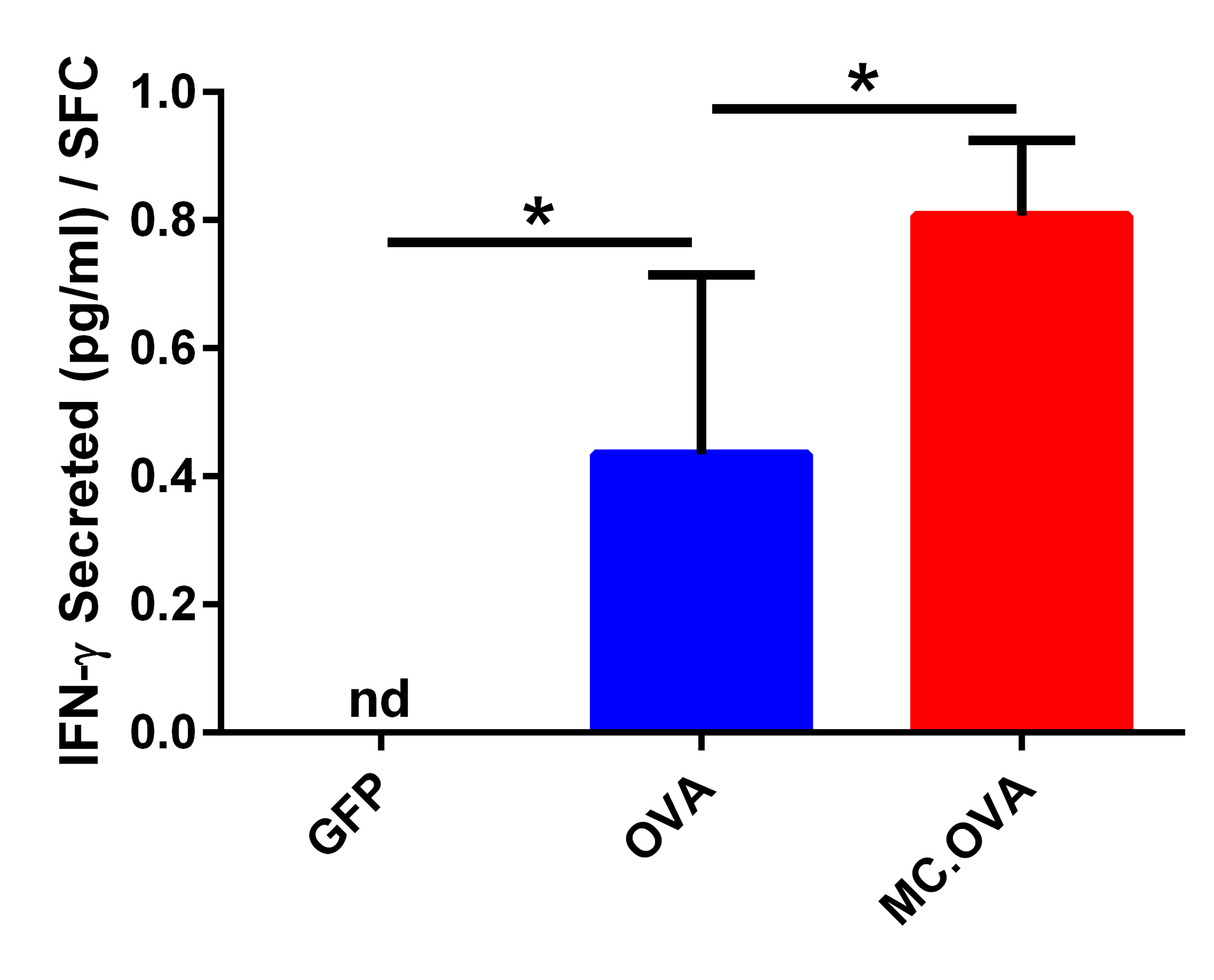

Supplement: S2 Fig — Naïve mice were vaccinated on day 0 and again 20 days later with 25 μg of plasmid DNA followed by electroporation. Some mice were injected with 1.25 mg/kg IP the day following each vaccination. On day 27 splenocytes were isolated from the mice and analyzed for Ag-specific T cells by (A) IFN-γ ELISpot and levels of IFN-γ secretion from SIINFEKL stimulated splenocytes, were quantified by ELISA. (B) ELISA-measured IFN-γ pg/ml was divided by SFC values for each replicate and the values plotted to give an estimate of the amount of IFN-γ secreted by each SIINFEKL-specific T cell. Values for MC.OVA ± rim were pooled. Analysis by One-Way ANOVA with Tukey correction for multiple comparisons, n = 5–10, *p<0.05 (TIF) [file pone.0164547.s002.tif]

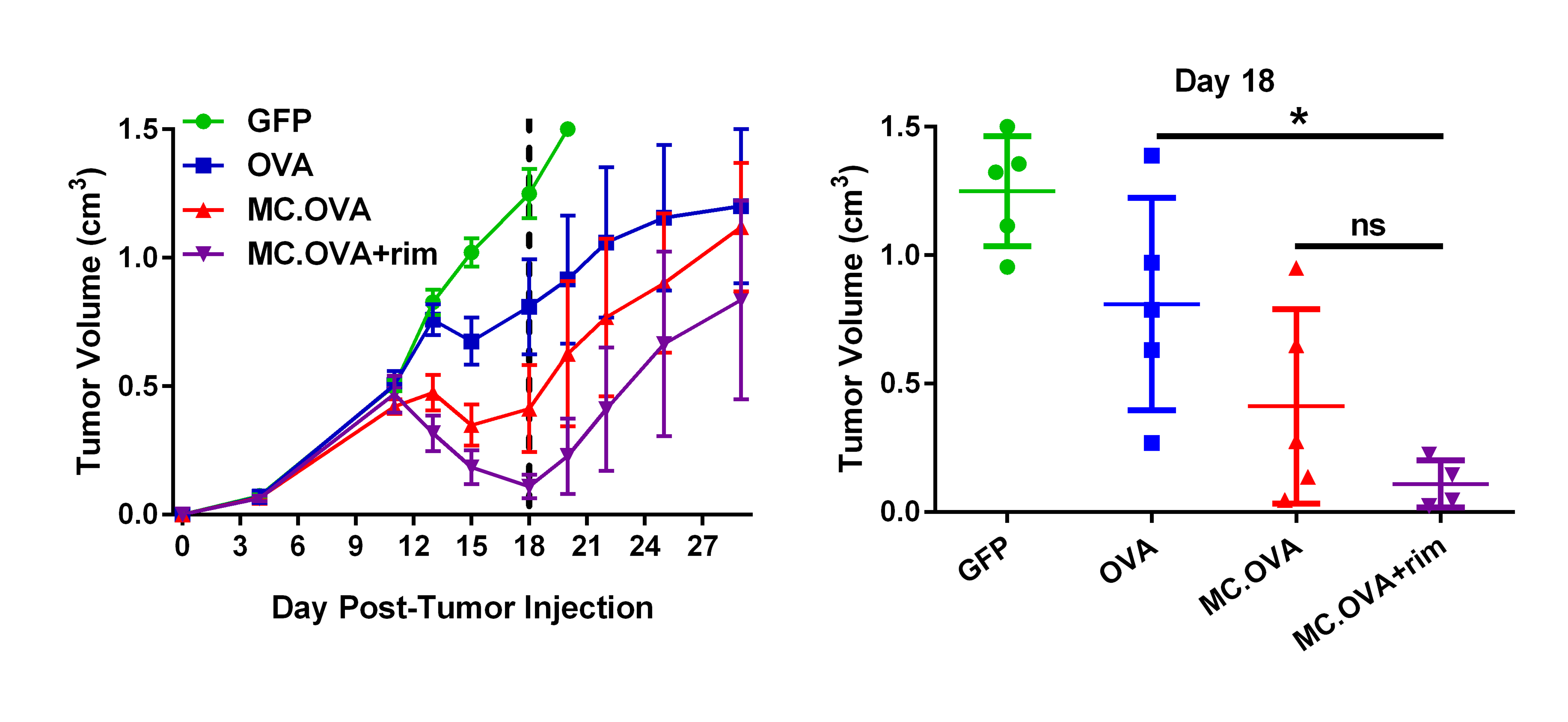

Supplement: S3 Fig — Mice were injected subQ with 1 x 10^6^ E.G7 cells on day 0. Mice were randomized on day 4 to normalize inter-group tumor volume. On days 5, 11, and 21 mice were vaccinated with 25 μg of the indicated plasmid in alternating flanks by EP. One day following each vaccination, 1.25 mg/kg rim was administered in MC.OVA + rim-treated mice. Tumor volumes were measured using calipers and the following equation; Volume (cm^3) = (0.5236) x L x W^2. Dotted lines in left hand panel indicate the time point at which groups were compared in the right panel. n = 4–5, *p<0.05, Analysis by One-Way ANOVA with Tukey correction for multiple comparisons. (TIF) [file pone.0164547.s003.tif]

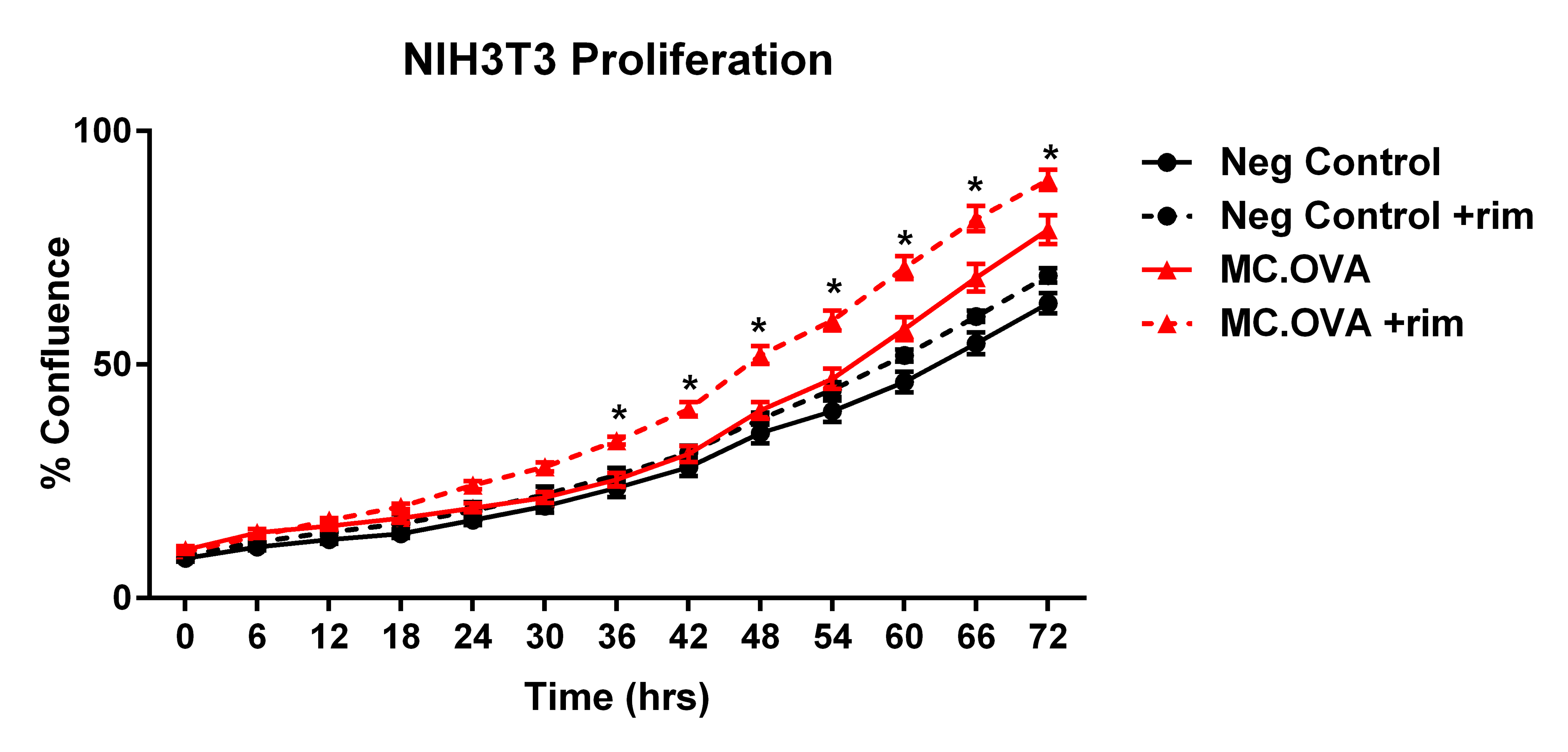

Supplement: S4 Fig — 2.5 x 10^5^ of either negative control or MC-modified NIH3T3 FBs were plated into a 96-well flat-bottom plate. The wells were then imaged at 6 hour intervals using an IncuCyte live cell analysis system. Images were analyzed for percent confluency of bright field well-images. n = 6, *p<0.05 compared to Neg Control +rim, Two-way ANOVA with repeated measures and Tukey correction for multiple comparisons. (TIF) [file pone.0164547.s004.tif]

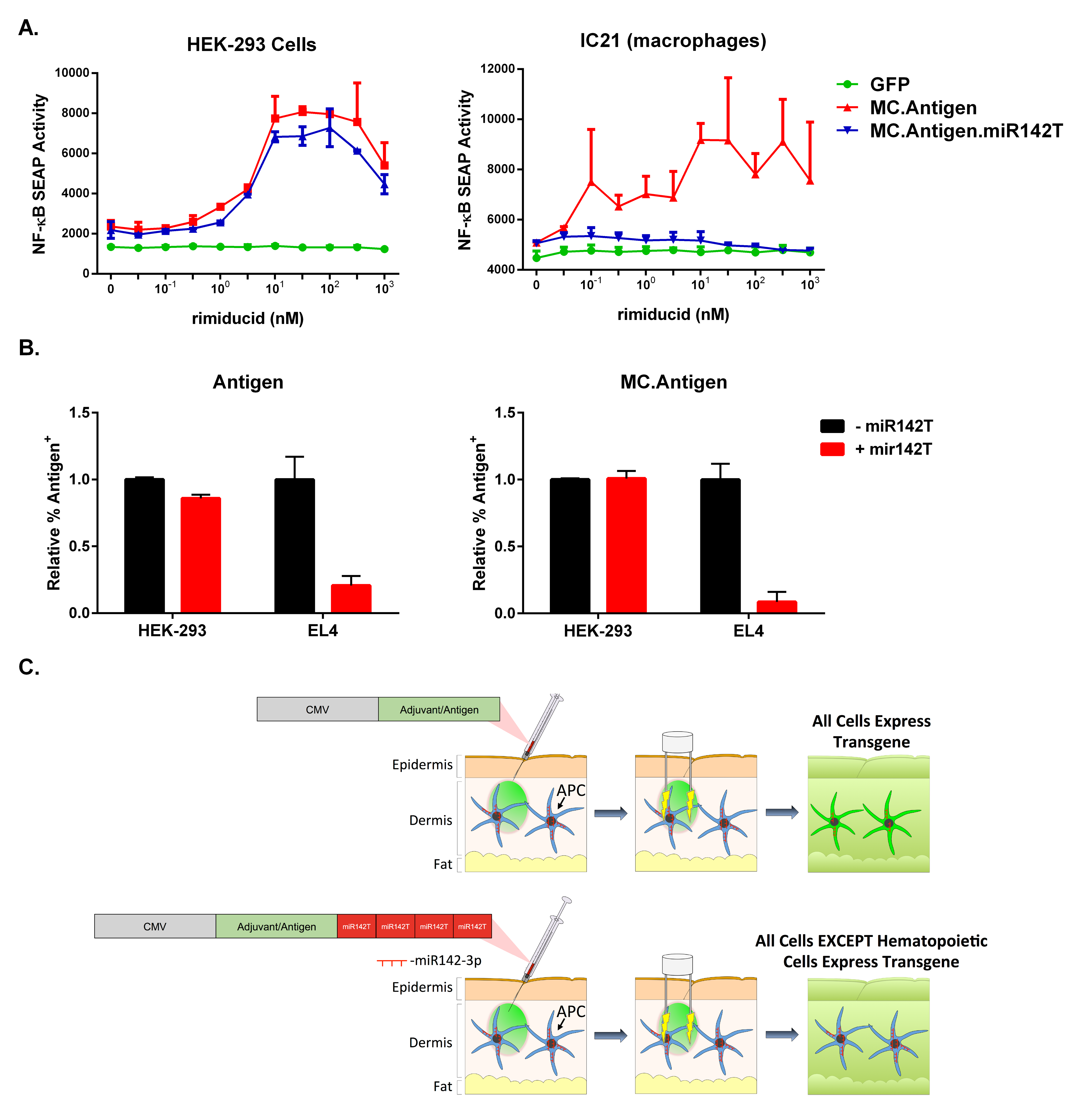

Supplement: S5 Fig — (A) Non-hematopoietic HEK-293 or hematopoietic IC21 cells were cotransfected with NF-κB SEAP reporter and either GFP, MC.Antigen (MC.PSMA), or MC.Antigen.miR142T (MC.PSMA.miR142T). Transfected cells were plated with dilutions of rimiducid. SEAP activity was assayed after 24 hours. (B) Non-hematopoietic HEK-293 cells were transfected or hematopoietic EL4 cells were nucleofected with a plasmid expressing either Antigen (PSMA, Left panel) or MC.Antigen (MC.PSMA, Right panel) with or without the miR142T sequence. After 24 hours Ag (PSMA) expression was assessed by flow cytometry. Values relative to corresponding -miR142T vector transfected cells. (C) Top Panel: EP of parental vectors results in global expression of transgene in all cell types at the site of administration, including APCs, as indicated by the green. Bottom Panel: EP of vaccine vectors containing miR142T miRNA target sequence prevent expression of vaccine-encoded proteins in cells differentiated from a hematopoietic lineage (e.g., DCs and macrophages), however expression in other cells types (e.g., keratinocytes) is still permitted. (TIF) [file pone.0164547.s005.tif]

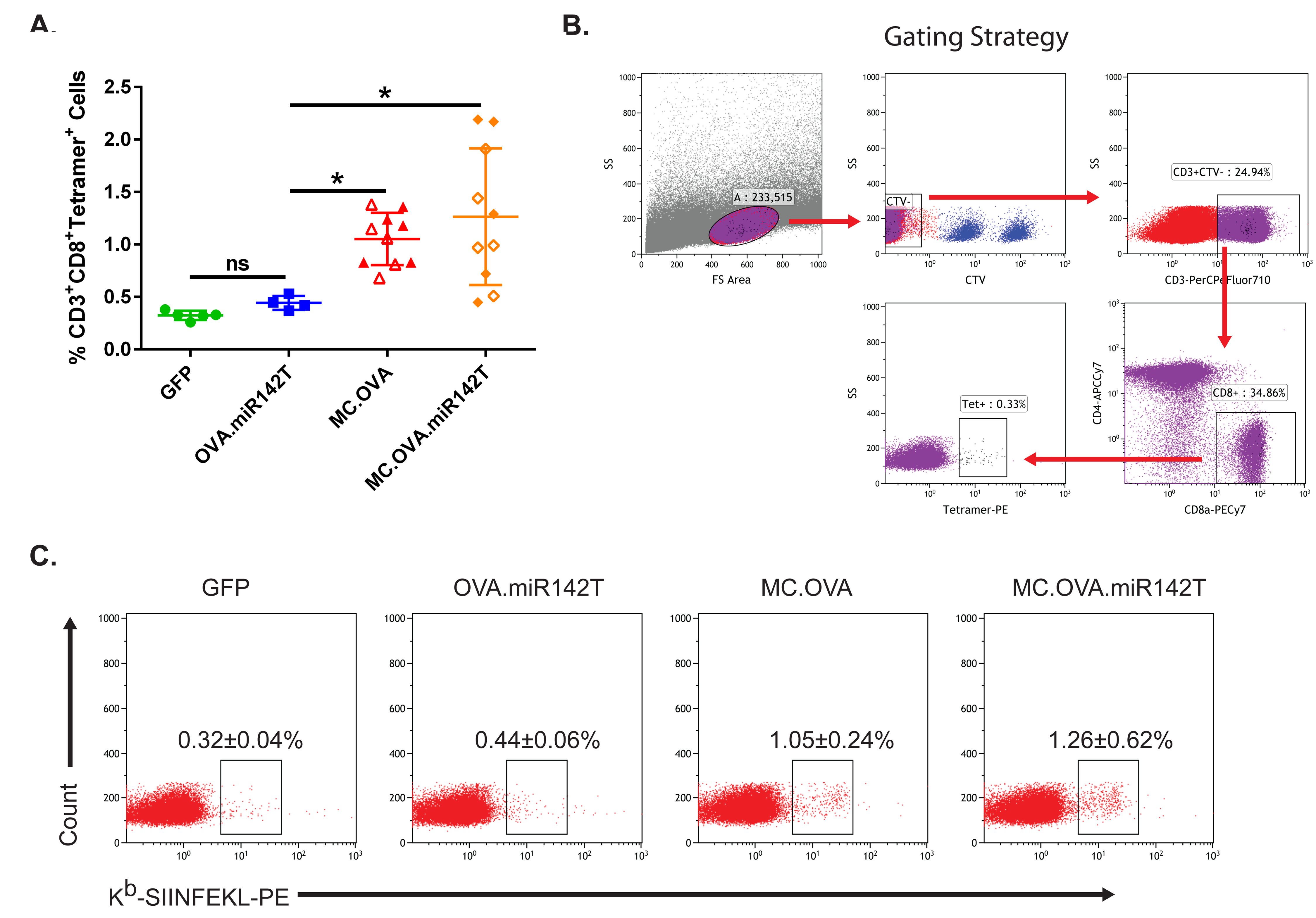

Supplement: S6 Fig — C57BL/6 mice were vaccinated on days 0 and 21 with 25 μg pDNA by EP. Some mice received rim, administered 1.25 mg/kg IP, the day following each vaccination. On day 28, 7 hours prior to termination, syngeneic splenocytes were adoptively transferred into mice for an in vivo CTL assay (Fig 8A and 8B). (A) Splenocytes were extracted 7 days after the final vaccination (day 28) and analyzed for H2-Kb-SIINFEKL Tetramer+ CD3+CD8+ T cells. (B) Gating strategy to remove adoptively transferred splenocytes by CTV. (C) Representative scatter plots for each group. Percentages are mean values ± SD. n = 5, *p<0.05, One-Way ANOVA with Holm-Šidák correction for multiple comparisons to OVA. (TIF) [file pone.0164547.s006.tif]
