# Supplementary material for: MyD88/CD40 Genetic Adjuvant Function in Cutaneous Atypical Antigen-Presenting Cells Contributes to DNA Vaccine Immunogenicity
Source: PLoS One. 2016 Oct 14;11(10):e0164547. doi: 10.1371/journal.pone.0164547 (PMC5065236; doi:10.1371/journal.pone.0164547)
Supplement: S1 Supplemental Methods — (DOCX) [file pone.0164547.s007.docx]

Supplemental Methods

*In vivo* electroporation and vaccination

*In vivo* electroporation (EP) was performed with a BTX ECM-2001 unit (Harvard Apparatus, Holliston, MA) for data reported in Figure S1A and C. All plasmid DNA used for *in vivo* applications was prepared using an endotoxin-free (EndoFree) Giga-prep kit (Qiagen, Hilden, Germany), and stock plasmid solutions were diluted in sterile PBS for a total volume of 50 μL per injection just prior to subcutaneous (subQ) administration. Animals were anesthetized using isoflurane during all procedures. Animals were shaved on their hind limbs and dorsal surfaces. 50 μL plasmid DNA (pDNA), 25-50 μg per injection, was injected intradermal (ID) into either the hind limb (BTX EP device). Immediately following injection, needle array probes were placed over the “bubble” formed by the injection bolus and electrical stimulation was applied. Settings for the BTX device were 6 pulses, 75 V, 20 ms duration, at 200 ms intervals. Initial vaccinations and subsequent booster vaccinations were administered on alternating, contralateral flanks.

For vaccinations that include the LacZ gene, animals were vaccinated once with a total DNA content of 50 μg, based on two distinct 25 μg injections, one on each hind limb. For vaccinations using OVA as the antigen in naïve mice, animals received two 25 μg doses of vaccine 14-21 days apart, each administered on contralateral flanks. Animals in groups receiving dimerizer were injected IP with 1.25 mg/kg rim 24 hours after each vaccination.

*In vivo* luciferase reporter assay

BALB/c mice were first injected with 50 μg FFLuc reporter plasmid and electroporated in their left hind limb. 50 μg of FFLuc reporter plasmid was then injected into the right hind limb of the same animals. Luciferase activity was measured using an IVIS imaging system (Perkin Elmer, Waltham, MA), following the manufacturer’s suggested protocols. Briefly, 100 μL D-luciferin (15 mg/mL stock solution in PBS) was injected IP in anesthetized animals 10 minutes prior to imaging. Luminescence data was analyzed using Living Image software (Perkin Elmer).

Draining lymph node analysis following EP

Mice were injected in both flanks with 50 μL PBS vehicle or with vehicle–resuspended 25 μg GFP or OVA pDNA with or without EP. 24 hours after treatment, lymphocytes from the inguinal lymph nodes were isolated. Lymph nodes were incubated in RPMI media with collagenase (1 mg/mL) for 30 minutes at 37°C. Following enzymatic digestion, single-cell suspensions were made by pipetting samples up and down, followed by filtration through a 70-μM strainer. Total live cells recovered were counted using a Cellometer with AOPI dye (Lawrence, MA). To identify the relative ratios of cell subsets, cell suspensions were stained with anti-mouse-CD3-FITC (Cat# 100204), anti-mouse-CD19-PE (Cat# 115508), and anti-mouse-CD11c-APC/Cy7 (Cat# 117324) (BioLegend, San Diego, CA) and analyzed on a Gallios flow cytometer using Kaluza® flow analysis software, version 1.3 (Beckman Coulter, Brea, CA).

NIH3T3 proliferation kinetics

In a 96-well plate 2 x 10^5^ of either negative control or MC.OVA transgenic NIH3T3 cells were seeded with or without 10 nM rim. Plates were incubated at 37^o^C in the IncuCyte living imaging system (Essen Biosciences, Ann Arbor, MI). Bright field images were taken at 6 hour intervals. Confluency was analyzed at each time point using IncuCyte Zoom software.

Analysis of PSMA antigen expression *in vitro*

293 cells were transfected in a 12-well plate with 1 μg of either PSMA, PSMA.miR142T, MC.PSMA or MC.PSMA.miR142T. EL4 cells were nucleofected with 2 μg of either PSMA, PSMA.miR142T, MC.PSMA or MC.PSMA.miR142T using an Amaxa 4D Nucleofector device and SF Cell Line kit (Lonza, Basal, Switzerland). After 24 hours, cells were harvested and stained with PE-conjugated anti-human PSMA antibody (Cat # 342403, Biolegend) and analyzed by flow cytometry for PSMA expression.
